# Nop9 is a PUF-like protein that prevents premature cleavage to correctly process pre-18S rRNA

**DOI:** 10.1038/ncomms13085

**Published:** 2016-10-11

**Authors:** Jun Zhang, Kathleen L. McCann, Chen Qiu, Lauren E. Gonzalez, Susan J. Baserga, Traci M. Tanaka Hall

**Affiliations:** 1 Epigenetics and Stem Cell Biology Laboratory, National Institute of Environmental Health Sciences, National Institutes of Health, PO Box 12233, MD F3-05, Research Triangle Park, North Carolina 27709, USA; 2 Department of Genetics, Yale University School of Medicine, New Haven, Connecticut 06520, USA; 3 Department of Therapeutic Radiology, Yale University School of Medicine, New Haven, Connecticut 06520, USA; 4 Department of Molecular Biophysics and Biochemistry, Yale University School of Medicine, New Haven, Connecticut 06520, USA

## Abstract

Numerous factors direct eukaryotic ribosome biogenesis, and defects in a single ribosome assembly factor may be lethal or produce tissue-specific human ribosomopathies. Pre-ribosomal RNAs (pre-rRNAs) must be processed stepwise and at the correct subcellular locations to produce the mature rRNAs. Nop9 is a conserved small ribosomal subunit biogenesis factor, essential in yeast. Here we report a 2.1-Å crystal structure of Nop9 and a small-angle X-ray-scattering model of a Nop9:RNA complex that reveals a ‘C'-shaped fold formed from 11 Pumilio repeats. We show that Nop9 recognizes sequence and structural features of the 20S pre-rRNA near the cleavage site of the nuclease, Nob1. We further demonstrate that Nop9 inhibits Nob1 cleavage, the final processing step to produce mature small ribosomal subunit 18S rRNA. Together, our results suggest that Nop9 is critical for timely cleavage of the 20S pre-rRNA. Moreover, the Nop9 structure exemplifies a new class of Pumilio repeat proteins.

## Abstract (web summary)

Nop9 is a conserved small ribosomal subunit biogenesis factor. Here, Zhang *et al*. show that Nop9, in complex with RNA, adopts a C-shaped fold formed from 11 Pumillo repeats and propose that Nop9 inhibits premature cleavage of 20S pre-rRNA by inhibiting the Nob1 nuclease.

## Graphical abstract

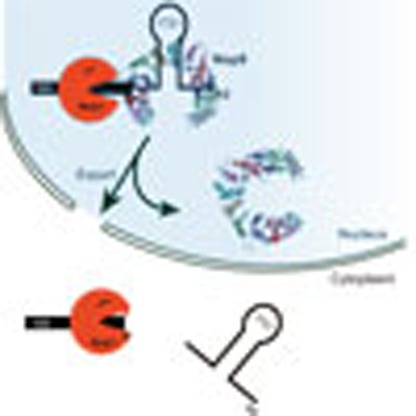

Ribosome biogenesis is a complex process wherein ribosomal RNAs (rRNAs) and ribosomal proteins are assembled to generate a large ribonucleoprotein complex for protein synthesis^1^,^2^,^3^,^4^. The pre-ribosomal RNA (pre-rRNA) undergoes multiple cleavage and trimming steps to remove the external and internal transcribed spacers and generate the mature rRNAs. Decades of studies have clarified the pathways of pre-rRNA processing and identified over 200 biogenesis factors. However, it is not known how many of these proteins facilitate proper processing of the pre-rRNA. One outstanding example is the cleavage step at site D of the 20S pre-rRNA to generate the mature 18S rRNA (Fig. 1a). Although cleavage occurs in the cytoplasm, Nob1, a nuclease that cleaves site D, is associated with the 20S pre-RNA in the nucleolus^5^,^6^,^7^,^8^,^9^,^10^. It is a mystery how Nob1 cleavage at site D is prevented before reaching the cytoplasm.

Nop9, a nucleolar protein conserved in humans, plants and yeast, is essential for 18S rRNA maturation^11^,^12^. Depletion of Nop9 in the yeast *Saccharomyces cerevisiae* abolishes synthesis of the small ribosomal subunit and therefore is lethal^11^. During pre-rRNA processing in the nucleolus, cleavage of the 32S pre-rRNA at site A2 generates the 27SA2 large ribosomal subunit pre-rRNA, precursor for the 25S and 5.8S rRNAs and the 20S small ribosomal subunit pre-rRNA (Fig. 1a). The 20S pre-rRNA is released from the nucleolus to the nucleoplasm and is then exported to the cytoplasm where cleavage at site D by Nob1 produces the mature 18S rRNA^5^,^6^,^10^. Previous studies suggested that Nop9 is important for 20S processing. Nop9 was shown to be associated with the small subunit (SSU) processome/90S pre-ribosome and the 20S pre-rRNA *in vivo*^9^,^11^,^13^. When Nop9 is depleted, the 20S pre-rRNA and 18S rRNA levels decrease, and pre-ribosomal particles whose pre-rRNAs retain the D-A2 sequence accumulate in the nucleolus^11^,^12^. The functional importance of Nop9 appears to be conserved in humans, as mutation of NOP9 is linked to a language-learning impairment, a ribosomopathy^14^.

Nop9 represents one of three distinct subfamilies of Pumilio/*fem-3* mRNA-binding factor (PUF) proteins, along with the classical PUF and the Puf-A/Puf6 subfamilies. Classical PUFs feature eight Pumilio (PUM) repeats arranged in a crescent shape^15^,^16^. By recognizing single-stranded RNA sequences with the concave surface^17^, classical PUF proteins regulate mRNA translation and stability^18^,^19^,^20^. Distinct from classical PUFs, Puf-A/Puf6 subfamily proteins are involved in large ribosomal subunit biogenesis, adopt an L-shaped structure comprising 11 PUM repeats and bind single- or double-stranded nucleic acids without apparent sequence specificity^12^,^21^. The divergence between classical PUF proteins and Puf-A/Puf6 proteins suggests that the subfamilies of PUF proteins have distinct cellular functions, protein folds and RNA-binding preferences. Knowledge of the three-dimensional structure of the Nop9 subfamily and identification of target RNAs would provide insight into the evolution of the PUF protein subfamilies as well as guide the understanding of Nop9's functions in ribosome biogenesis.

To reveal the relationship between atomic structure and function in ribosome biogenesis, we determined a 2.1-Å crystal structure of yeast Nop9 and probed its activity *in vitro* and *in vivo*. Distinct from the two other PUF protein subfamilies, Nop9 adopts a C-shaped fold containing 11 PUM repeats. Consistent with a role in processing 20S pre-rRNA to mature 18S rRNA, we find that Nop9 binds to the fragment of internal transcribed spacer 1 (ITS1) present in the 20S pre-rRNA. Different from the classical PUFs and Puf-A subfamilies, Nop9 binds specifically to ITS1 RNA fragments that encompass both single-stranded and duplex regions. A small-angle X-ray-scattering (SAXS) model of the Nop9:ITS1 RNA complex and biochemical probing confirm that Nop9 recognizes the base of the ITS1 RNA stem loop. Since the Nop9-binding site overlaps the binding site of Nob1, which processes 20S pre-rRNA to 18S rRNA in the cytoplasm, we examined whether Nop9 may play a role in stabilizing the 20S pre-rRNA in the nucleolus. We demonstrate that the levels of the 20S pre-rRNA are reduced upon depletion of Nop9 and restored to normal levels upon depletion of both Nop9 and Nob1 *in vivo*. Moreover, the presence of Nop9 reduces the cleavage efficiency of Nob1 *in vitro*. Together, our results suggest that Nop9's essential role in SSU ribosome biogenesis is to prevent premature cleavage of the 20S pre-rRNA by Nob1 in the nucleolus.

## Results

### Nop9 is a C-shaped PUF-like protein with 11 PUM repeats

**Figure 1:**
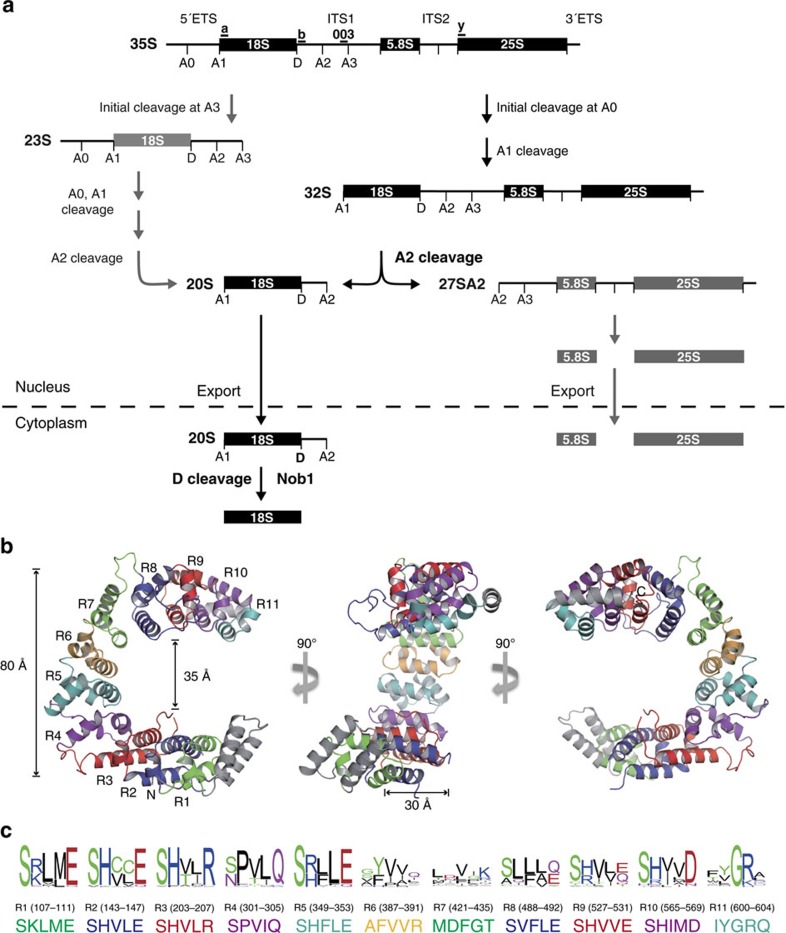
Crystal structure of *S. cerevisiae* Nop9 protein. (**a**) Diagram of pre-rRNA-processing steps in yeast. The pre-rRNA is transcribed as a 35S polycistronic precursor and is processed through a series of cleavage events to produce the mature 18S, 5.8S and 25S rRNAs. Oligonucleotide probes, 003, a, b and y for northern blotting are indicated. The 20S pre-rRNA is produced by cleavage at site A2, and after export to the cytoplasm, it is cleaved at site D to produce the mature 18S rRNA. (**b**) Ribbon drawing of Nop9. The 11 PUM repeats are coloured sequentially and denoted R1 through R11. N- and C-terminal capping helices are shown in grey. Three orientations are shown, rotated 90° with respect to one another. The molecular graphics for this figure (Figs 4 and 7) and Supplementary Figs 1 and 4 were prepared with PyMol (Schrödinger). (**c**) Conservation of Nop9 RNA-recognition motifs. The sequence logos of putative RNA-binding motifs in Nop9 family members (top) and the residue numbers (middle) and corresponding sequences (bottom) in *S. cerevisiae* Nop9 are shown. The sequence logos were generated by WebLogo^52^ using 108 Nop9 family sequences from different organisms. The alignment was carried out using ClustalX 2.1.

We determined a 2.1-Å-resolution crystal structure of yeast Nop9 that revealed a new PUM repeat fold with 11 PUM repeats (R1 to R11) arranged in a C-like shape (Fig. 1b, Supplementary Fig. 1a and Table 1). The repeats form a twisted, incomplete ring with an ∼80-Å outer diameter, an ∼35 Å inner diameter and 30–40 Å thickness. Most of the individual PUM repeats are similar to classical PUM repeats with three *α* helices, but with variability in the length of helices and interhelix loops (Supplementary Fig. 1b,c). The repeats form a curved overall structure, and twisting relative to imaginary repeat-to-repeat axes is focused at repeats R4 and R7 (Supplementary Fig. 1d). Most of the variable interhelix regions correspond to these locations where twisting occurs. Extended loops before and after repeat R4 and a long loop between repeats R7 and R8 each correspond to ∼25° twists. Repeat R11 diverges in structure from a classical PUM repeat with two α helices at the C-terminal end that cap the structure. Two long loops between helices α2 and α3 in repeats R2 (residues 164–174) and R3 (residues 220–250) are disordered in the crystal structure (Supplementary Fig. 2).

The most-conserved residues across Nop9 family proteins are found on the inner concave surface at the positions that typically recognize RNA bases in classical PUF proteins (Fig. 1c). These five-residue motifs are found near the N-terminal ends of the α2 helices (Supplementary Fig. 2). The most common motif in Nop9, SϕxxE/D (where ϕ is a stacking side chain and x is a hydrophobic side chain), specifies a G base in classical PUF proteins such as PUM1. This motif is found in repeats R1, R2, R5 and R8-R10 of Nop9. A C-recognition motif, sϕxxR (where s is a small side chain and ϕ is a stacking side chain), is present in repeats R3 and R6 of Nop9, although the motif in R6 is not conserved among the Nop9 family (Fig. 1c). In contrast to the divergent five-residue motifs of Puf-A family proteins^21^, most of Nop9's PUM repeats bear motifs similar to the sequence-specific PUM repeats of classical PUF proteins^17^,^22^. We thus sought to identify an RNA target sequence for Nop9 in pre-rRNA.

### Nop9 recognizes the sequence and structure of ITS1 pre-rRNA

**Figure 2:**
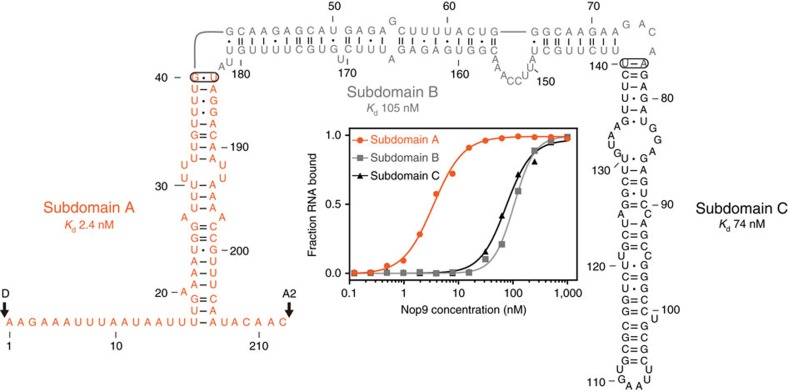
Nop9 binds specifically to the ITS1 D-A2 fragment of 20S pre-rRNA. The secondary structure of the ITS1 D-A2 pre-rRNA (1–212) is shown. Watson–Crick base pairs are denoted by single (A–U) or double (G–C) dashes, and G–U wobble base pairs are denoted by dots. We divided the ITS1 D-A2 pre-rRNA into three stem–loop subdomains: A (orange), B (grey) and C (black). The ends of subdomains A and B were closed by connecting nucleotides 40 and 184 or nucleotides 77 and 140, respectively (ovals). Representative binding curves for Nop9 and ITS1 D-A2 subdomains A, B and C are shown (inset). The mean *K*_d_ values for three technical replicate experiments are noted, and the mean *K*_d_±s.e.m. are summarized in Supplementary Table 1.

We used electrophoretic mobility shift assays (EMSAs) and found that Nop9 binds to ITS1 pre-rRNA near site D, the site cleaved by Nob1 to produce the mature 18S rRNA from the 20S pre-rRNA. Previously, Nop9 had been shown to bind *in vitro* to an immobilized fragment of ITS1 from site D to site A2 (D-A2) at the 3′ end of 20S rRNA^11^. However, Nop9 also associated with immobilized tRNA, suggesting a lack of specificity. Therefore, to identify a pre-rRNA-binding site for Nop9, we began by measuring binding affinities of Nop9 for three subdomains of the D-A2 fragment of ITS1 (labelled A, B and C in Fig. 2). We found that Nop9 bound with strong affinity to subdomain A (*K*_d_=2.4±0.3 nM, ±indicates s.e.m., *n*=3), which contains the 5′ and 3′ single-stranded flanking regions and the base of a long stem loop (Fig. 2, Supplementary Fig. 3 and Supplementary Table 1). Nop9 binds 30–40-fold more weakly to Subdomain B or C.

We next sought to narrow the Nop9-binding site within Subdomain A and found that the base of the stem–loop region and the adjacent 5′ flanking region were critical for Nop9 binding. The 5′ single-stranded nucleotides 1–16 were necessary, but are not sufficient for binding: deleting 5′ nucleotides 1–16 abrogated binding, but nucleotides 1–16 alone did not bind Nop9 (Supplementary Table 1). We could trim 5′ nucleotides 1–6 without affecting binding affinity, indicating the importance of 5′ nucleotides closer to the stem. Deleting the 3′ single-stranded region reduced binding affinity 6.5-fold, suggesting that it contributes to binding affinity, but not to the same extent as the 5′ flanking nucleotides and stem loop. Classical PUF proteins such as PUM1 recognize RNA bases using hydrogen bond and stacking interactions, and binding is insensitive to salt concentration^17^. In contrast, interaction of Puf-A/Puf6 proteins with phosphate groups of single- or double-stranded RNA is highly sensitive to salt concentration^21^. Nop9:ITS1 RNA binding was only modestly sensitive to salt concentration (Supplementary Table 2), consistent with specific H-bond or base-stacking interactions that recognize RNA sequence elements.

**Figure 3:**
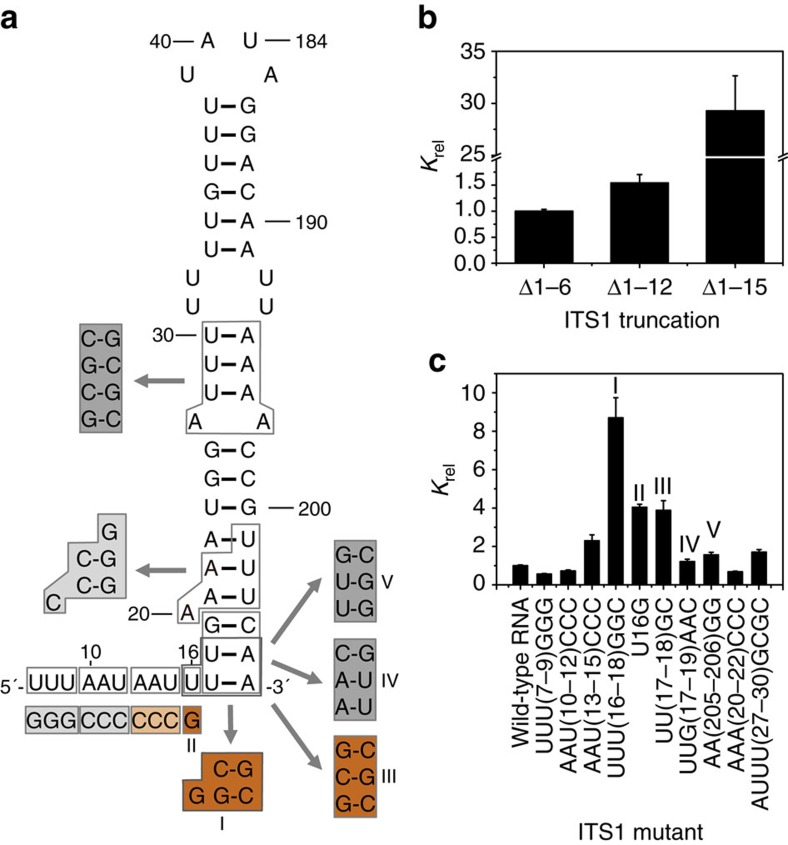
Nop9 recognizes sequence and structural elements of ITS1 RNA. (**a**) RNA sequence and secondary structure of ITS1 subdomain A. Mutations tested in **c** are indicated by the boxed sequences and coloured according to the effect on relative *K*_d_ (*K*_rel_). *K*_rel_ was set equal to 1 for Nop9 binding to the ITS1 subdomain A Δ5′_1–6_, Δ3′ (mean *K*_d_±s.e.m.=11.8±0.4 nM for four technical replicate experiments). *K*_rel_>3, dark orange; 2<*K*_rel_<3, light orange; 1<*K*_rel_<2, dark grey; *K*_rel_≤1, light grey. As noted in the text, the mutation at the base of the stem region (I) was probed further with additional mutants (II, III, IV and V). (**b**) RNA nucleotides 13–15 are critical for Nop9 binding to ITS1 subdomain A. A bar graph created in GraphPad PRISM plots the mean binding affinities of Nop9 and 5′ truncated ITS1 subdomain A RNAs relative to Nop9 binding to ITS1 subdomain A Δ5′_1–6_, Δ3′ calculated from three technical replicate experiments with error bars representing the s.e.m. (**c**) RNA nucleotides 13–18 are important for Nop9 binding to ITS1 subdomain A. A bar graph created in GraphPad PRISM plots the mean binding affinities of Nop9 and mutated ITS1 RNAs relative to Nop9 binding to ITS1 subdomain A Δ5′_1–6_, Δ3′ calculated from three technical replicate experiments with error bars representing the s.e.m. *K*_rel_ for Nop9 and ITS1 mutants are labelled as in **a**. For mutations in the stem region, base pairing was maintained by corresponding mutations to the opposite strand. The mean *K*_d_±s.e.m., *K*_rel_, and *P* values are summarized in Supplementary Table 3.

We systematically mutated an ITS1 pre-rRNA fragment and found that Nop9 is selective for both the sequence and structure of ITS1 (Fig. 3 and Supplementary Table 3). We first analysed the importance of the 5′ flanking sequence, whose deletion abrogated binding. Trimming up to 12 nucleotides from the 5′ single-stranded region had a minor effect on binding affinity (Fig. 3b); similarly, mutating the RNA sequence in the distal part of the 5′ flanking region (nucleotides 7–9 and 10–12) had little to no effect on Nop9 binding (Fig. 3c). In contrast, removing 15 nucleotides from the 5′ single-stranded region diminished binding affinity 29-fold (Fig. 3b), and sequence mutations nearing the base of the duplex (nucleotides 13–15) reduced RNA-binding affinity (Fig. 3c). We next analysed the importance of the sequence of the duplex region for Nop9 binding and found that the lower stem and base U16 are critical for RNA-binding affinity. Mutating nucleotides 16–18 from UUU to GGC while preserving base pairing (Figs 3a, I) reduced affinity approximately ninefold (Fig. 3c). We separated the U16G mutation in the 5′ flanking sequence (Fig. 3a, II) from the UU17-18GC mutation (Fig. 3a, III), which switches the two U–A base pairs at the base of the stem to G–C base pairs, and found that either mutation reduced RNA-binding affinity approximately fourfold (Fig. 3c). Mutation of U16 to A or C also weakened RNA-binding affinity (Supplementary Table 3). In contrast, reversing the sequence of the 5′ and 3′ strands at the base of the duplex (Fig. 3a, IV), which maintains U–A versus G–C base pairs, had no effect on Nop9 binding (Fig. 3c). Preference for RNAs with U–A or A–U versus G–C base pairs is not because of weaker hydrogen bonding because mutations that result in U–G base pairs at the base of the duplex reduce RNA-binding affinity approximately threefold (Fig. 3a, V). The sequence of the upper stem is not critical for Nop9 interaction; mutation of nucleotides 20–22 or 27–30 while maintaining base pairing had little effect on Nop9 RNA-binding affinity (Fig. 3c). Taken together, we found that Nop9 prefers uridine at position 16 in the 5′ single-stranded region of the ITS1 pre-rRNA and U–A or A–U base pairs at the base of the duplex region, suggesting that specific protein:RNA interaction may be focused in this region.

### Nop9 binds at the base of the ITS1 stem loop

**Figure 4:**
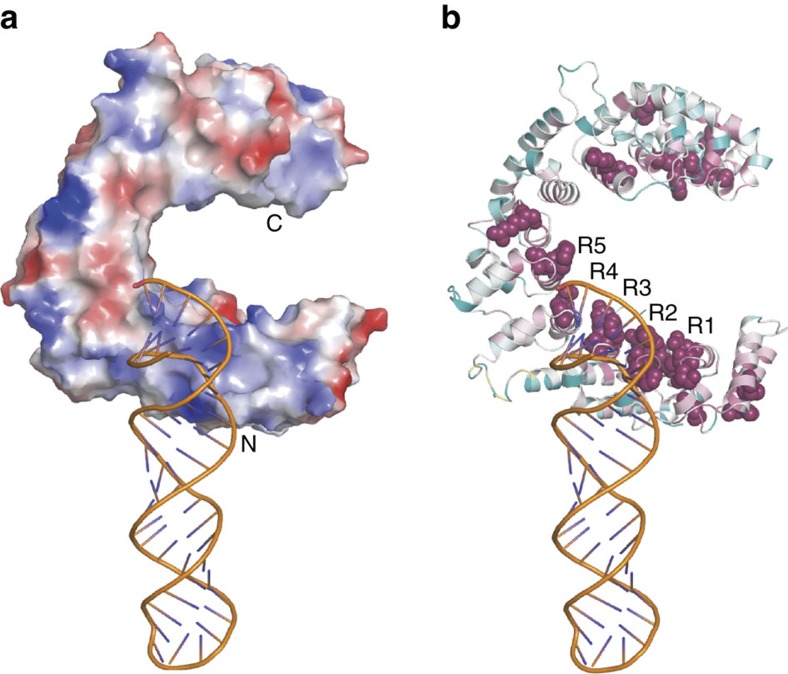
SAXS model of the Nop9 protein and ITS1 D-A2 subdomain A pre-rRNA. (**a**) Rigid body SAXS model of the Nop9:ITS1 D-A2 subdomain A pre-rRNA complex. The crystal structure of Nop9 and a predicted model of the ITS1 D-A2 Subdomain A pre-rRNA from SAXS data of the RNA alone are shown. Positively and negatively charged surfaces are shown in blue and red, respectively. The vacuum electrostatic surface potential was calculated using the APBS module within PyMol. (**b**) ITS1 D-A2 pre-rRNA contacts the conserved Nop9 repeats. The extent of amino-acid sequence conservation was calculated using the ConSurf server^53^ with default settings, and the results are displayed on a ribbon diagram of Nop9 (highly conserved residues are coloured maroon and less conserved residues are cyan). The most highly conserved residues are shown with space-filling spheres.

To build a Nop9:ITS1 RNA complex structure, we calculated a SAXS model of the complex that placed the open end of the RNA duplex near Nop9's N-terminal repeats. We collected SAXS data for Nop9 alone, ITS1 RNA (Subdomain A) and a Nop9:ITS1 RNA complex (Supplementary Fig. 4 and Supplementary Table 4). We first confirmed that the crystal structure of Nop9 matches the SAXS data for Nop9 alone and that a model for the ITS1 RNA matches that of the RNA alone. We then employed rigid body modelling with the Nop9 crystal structure and the ITS1 RNA model, assuming no large conformational changes to build a protein:RNA complex that best fits the SAXS data for the protein:RNA complex (Fig. 4 and Supplementary Fig. 4). The Nop9:ITS1 RNA SAXS model places the open end of the ITS1 duplex on the surface of Nop9 near the loops between the α1 and α2 helices of repeats R2 and R3. Indeed, a basic patch on this surface of Nop9 complements the negatively charged RNA phosphate backbone (Fig. 4a). In addition, the N terminus of Nop9 is near the major groove of the RNA (Fig. 4a). The sequence of the N terminus is highly conserved (Fig. 4b), suggesting a possible role in RNA interaction. The low resolution of SAXS modelling and the flexible nature of single-stranded RNA prevented visualization of the 5′ flanking RNA of ITS1.

### Nop9 impedes Nob1 cleavage in 20S pre-rRNA processing

**Figure 5:**
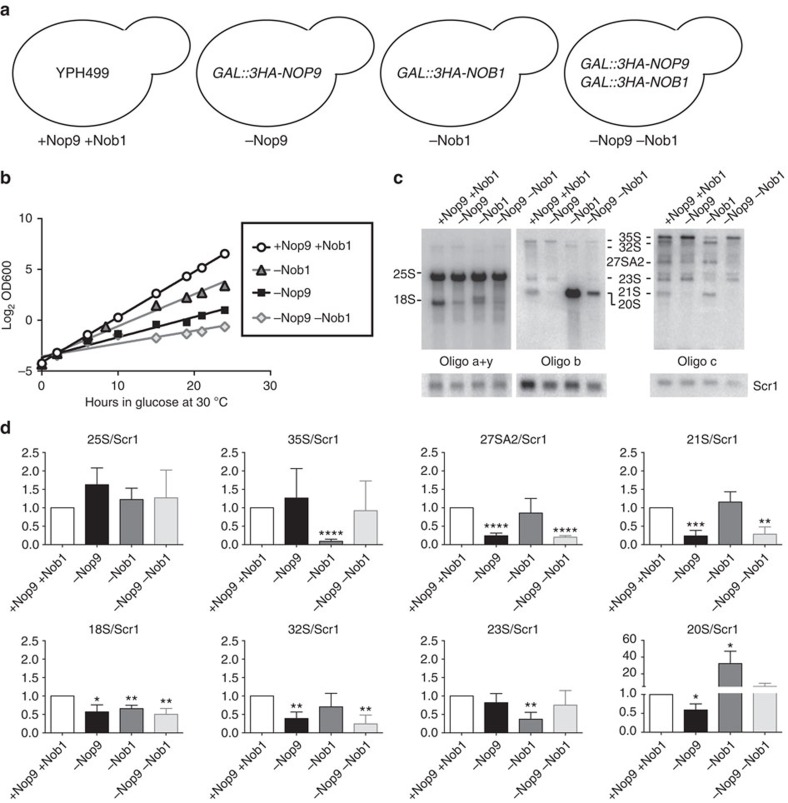
Probing the *in vivo* function of Nop9. (**a**) Schematic drawings of the yeast strains used for testing the effects of Nop9 and/or Nob1 depletion. Chromosomal Nop9 and/or Nob1 expression was placed under the control of the inducible *GAL* promoter and sequences encoding triple-HA tags were inserted. (**b**) Depletion of Nop9 and/or Nob1 expression impairs growth. Representative growth curves of yeast strains grown at 30 °C for 24 h in glucose to deplete endogenous Nop9 and/or Nob1 expression. Three biological replicates were conducted. (**c**) Northern blot analysis of Nop9/Nob1 double depletion indicates restoration of normal 20S pre-rRNA levels. Representative northern blots detecting precursor and mature rRNA in total RNA from parental YPH499 (+Nop9 +Nob1) or yeast depleted of Nop9 (−Nop9), Nob1 (−Nob1) or both Nop9 and Nob1 (−Nop9 −Nob1) in glucose for 24 h at 30 °C. The pre-rRNA intermediates and mature rRNAs were detected using a series of oligonucleotide probes, a, b, c and y, that are indicated in Fig. 1a. Oligos a+y were used to detect 25S and 18S rRNAs, oligo b was used to detect the 20S pre-rRNA and oligo c was used to detect the 35S, 32S, 27SA2, 23S and 21S pre-rRNAs. An oligo complementary to Scr1 was used as a loading control. (**d**) Quantitation of replicates of the northern blots presented in **c**. The intensities of the mature rRNAs and pre-rRNA intermediates relative to Scr1, the loading control, were plotted. Bar graphs created in GraphPad PRISM plot the mean intensities calculated from three biological replicate experiments with error bars representing the s.e.m. The significance of the levels of mature and pre-rRNAs in −Nop9, −Nob1 or −Nop9 −Nob1 compared with +Nop9 +Nob1 YPH499 was assessed by an unpaired, two-sided *t*-test, and *P* values are indicated (*P≤0.05, **P≤0.01, ***P≤0.001 and ****P≤0.0001 nonsignificant differences have *P* values >0.05). The analysis of the 35S, 32S and 23S pre-rRNAs using oligo c is shown; however, similar results are obtained when analysing the blot probed with oligo b.

The endonuclease Nob1 processes 20S pre-rRNA to 18S rRNA in the cytoplasm; however, its cleavage activity must be prevented in the nucleolus. Since Nob1's recognition site includes the base of the stem in ITS1 where we found that Nop9 binds^6^,^23^, we hypothesized that Nop9 might compete with Nob1 substrate recognition and consequently inhibit Nob1 cleavage of the 20S pre-rRNA at site D in the nucleolus. To test this hypothesis, we generated three distinct yeast strains where endogenous, chromosomal *NOP9*, *NOB1* or both *NOP9* and *NOB1* were encoded with a triple-haemagglutinin (HA) epitope tag and were placed under the control of a galactose-inducible, glucose-repressible promoter (Fig. 5a, −Nop9, −Nob1 and −Nop9 −Nob1). When these strains are grown in the presence of glucose, expression of Nop9, Nob1 or both Nop9 and Nob1 is inhibited and the protein is depleted (Supplementary Fig. 5a). Since both Nop9 and Nob1 are essential for small ribosomal subunit assembly, depletion of Nop9, Nob1 or both Nop9 and Nob1 should impair growth^5^,^11^. We assessed yeast growth rate after depletion of endogenous Nop9, Nob1 or both Nop9 and Nob1 for 24 h at 30 °C (Fig. 5b, −Nop9, −Nob1, −Nop9 −Nob1). As expected, depletion of Nop9 (−Nop9), Nob1 (−Nob1) or both Nop9 and Nob1 (−Nop9 −Nob1) impaired growth relative to the parental strain YPH499 (+Nop9 +Nob1)^11^.

To probe the *in vivo* role of Nop9 in preventing premature 20S pre-rRNA cleavage, we analysed the effects of Nop9, Nob1 or Nop9 and Nob1 depletion on pre-rRNA processing by northern blot analysis. We quantitated the changes in the abundance of the mature rRNAs or pre-rRNA intermediates relative to a loading control, Scr1 (Fig. 5c,d and Supplementary Fig. 5b). Depletion of Nop9 did not affect mature 25S rRNA levels, but did result in a significant decrease in mature 18S rRNA levels compared with the parental strain (Fig. 5c,d, +Nop9 +Nob1 versus −Nop9), consistent with its role in small ribosomal subunit assembly^11^,^12^. Nop9 depletion did not significantly affect the levels of the 35S or the 23S pre-rRNA intermediates, yet the levels of the 32S, 27SA2, 21S and 20S pre-rRNAs were decreased compared with the parental strain (Fig. 5c,d, +Nop9 +Nob1 versus −Nop9). These results are consistent with previous results that depletion of Nop9 affects the early U3-dependent cleavages at A0, A1 and A2 (ref. 11).

However, we also examined depletion of Nop9 for 72 h at 17 °C, an optimized temperature at which ribosome biogenesis defects are enhanced (Supplementary Fig. 6a)^24^,^25^. Under these conditions, cleavage at site A2, which generates the 20S pre-rRNA, does not appear to be dramatically inhibited, since the levels of 27SA2 and 23S pre-rRNA were only mildly affected by Nop9 depletion (Supplementary Fig. 6b,c), consistent with findings from a screen to identify additional ribosome biogenesis genes^12^. Yet, we observed a significant reduction in the levels of the 20S pre-rRNA relative to the loading control, Scr1 (Supplementary Fig. 6b,c). As a result, the decrease in the 20S pre-rRNA includes a defect in its further processing and/or stability, not only in its production. This is consistent with our hypothesis that Nop9 prevents premature cleavage of the 20S pre-rRNA in the nucleolus.

Depletion of Nob1 also impaired small ribosomal subunit assembly. The levels of the mature 18S rRNA but not the mature 25S rRNA were significantly decreased relative to the parental strain (Fig. 5c,d, +Nop9 +Nob1 versus −Nob1). Depletion of Nob1 resulted in a reduction of the 35S and the 23S pre-rRNA intermediates, while the levels of the 32S, 27SA2 or 21S pre-rRNA intermediates were not significantly affected. Strikingly, Nob1 depletion did result in a significant accumulation of the 20S pre-rRNA relative to the parental strain (Fig. 5c,d, +Nop9 +Nob1 versus −Nob1). This is consistent with previous results and demonstrates the importance of Nob1 for 20S pre-rRNA processing^5^.

On the basis of our hypothesis that Nop9 might compete with Nob1 substrate recognition and consequently reduce Nob1 cleavage at site D in the nucleolus, we expected that depleting Nob1 together with Nop9 *in vivo* would restore the levels of the 20S pre-rRNA. We found that co-depletion of Nop9 and Nob1 resulted in a SSU defect, as the levels of mature 25S rRNA were not affected, but the levels of mature 18S rRNA were significantly reduced (Fig. 5c,d, +Nop9 +Nob1 versus −Nop9 −Nob1). Similar to depletion of Nop9, co-depletion of Nop9 and Nob1 did not affect the levels of the 35S or the 23S pre-rRNAs, while the levels of the 32S, 27SA2 and 21S pre-rRNAs were reduced. Importantly, as predicted, although the 18S rRNA levels were diminished upon co-depletion of Nop9 and Nob1, the 20S pre-rRNA level was restored to the parental strain level (Fig. 5c,d, −Nop9 −Nob1 versus +Nop9 +Nob1). These *in vivo* observations support the proposal that Nop9 restricts Nob1 substrate recognition in the nucleolus.

**Figure 6:**
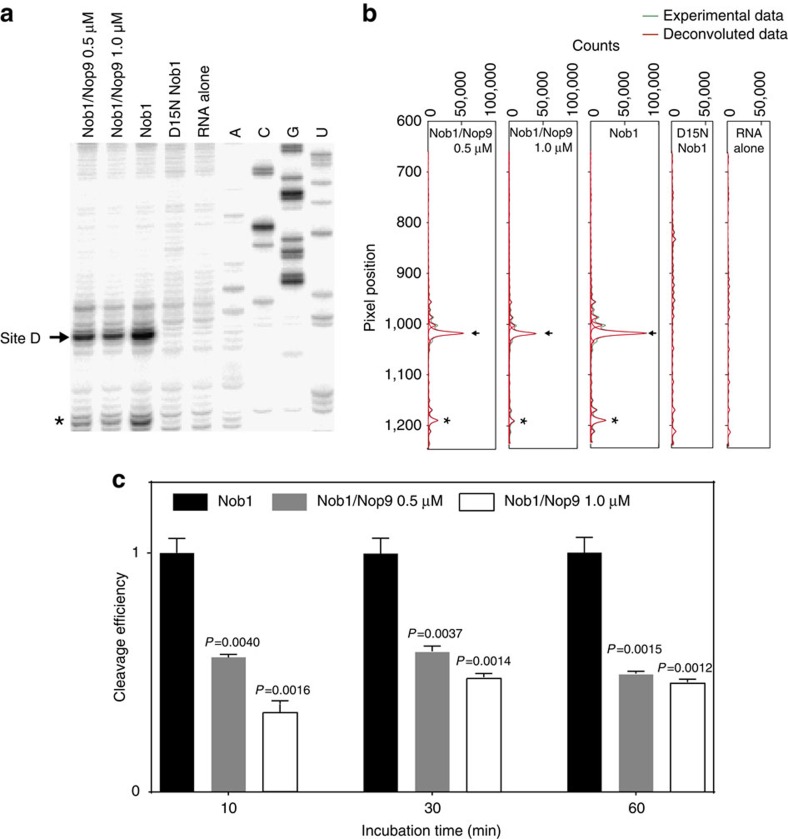
Nop9 reduces Nob1 cleavage efficiency. (**a**) Primer extension analysis of Nob1 cleavage. A pre-rRNA substrate containing the 3′ end of 20S pre-rRNA (1 μM) was incubated with Nob1 (3.5 μM) for 60 min in the presence or absence of Nop9 (0.5 or 1.0 μM) and products detected using primer extension analysis. Control reactions with a catalytically inactive Nob1 D15N mutant or RNA substrate alone are included. A representative gel is shown. An arrow indicates a major product with cleavage at site D. An asterisk indicates a minor product at an alternative cleavage site. Sequencing reactions are shown in the right lanes. The full gel is shown in Supplementary Fig. 6a. (**b**) Profiling of the sequencing gel lanes surrounding site D. SAFA was used to deconvolute the sequencing gel in **a**. (**c**) Nob1 cleavage efficiencies at different time points and concentrations of Nop9. Cleavage efficiencies were calculated as the ratio of site D cleavage product to total RNA and the efficiency of cleavage by Nob1 alone was set to 1 for each time point. The mean cleavage efficiencies±s.e.m. for Nob1 were 1.15±0.06% at 10 min, 3.54±0.23% at 30 min and 7.73±0.50% at 60 min. Bar graphs created in GraphPad PRISM plot the means calculated from three technical replicate experiments with error bars representing the s.e.m. The significance of the cleavage efficiencies in the presence of Nop9 relative to Nob1 cleavage alone was assessed by an unpaired, two-sided *t*-test, and *P* values are indicated.

To directly test whether Nop9 inhibits Nob1 cleavage of ITS1 RNA at site D, we performed an *in vitro* assay and demonstrated that Nop9 reduces Nob1 cleavage efficiency. We incubated a pre-rRNA substrate with purified Nob1 and used primer extension analysis to identify Nob1 cleavage products^8^. As was observed in previous studies^8^,^10^, we found a major product at cleavage site D, and the efficiency of *in vitro* Nob1 cleavage was modest (Fig. 6a,b and Supplementary Fig. 7a). Cleavage at site D was produced by Nob1 activity, not contaminating nuclease activity, as we did not observe this product with the catalytically inactive Nob1 mutant D15N (Fig. 6a,b and Supplementary Fig. 7a). We next measured the effect of Nop9 on Nob1 cleavage, and we detected reduced site D product in the presence of Nop9 after 10 min of reaction (Fig. 6c). Longer incubation times increased the efficiency of site D cleavage, and the presence of Nop9 reduced cleavage by ∼50%. This level of inhibition was reached with 1:1 or 0.5:1 molar ratios of Nop9 to the pre-rRNA substrate. To assure that inhibition was specific to Nop9, we confirmed that the presence of SUMO–Nop9 fusion protein reduced Nob1 site D cleavage efficiency by ∼50% relative to cleavage efficiency in the presence of SUMO alone (Supplementary Fig. 7b,c).

## Discussion

Pre-rRNA processing is a multistep process that requires a series of cleavages by endo- and exonucleases to generate mature rRNA transcripts. These cleavage steps occur within large ribonucleoprotein complexes whose component proteins and RNAs are dynamically changing. Pre-rRNAs also undergo modification and refolding to reach their mature forms, and ribosomal proteins and assembly factors associate with the pre-ribosome throughout its biogenesis. Our results here suggest that Nop9 plays an essential role in preventing Nob1 from cleaving pre-rRNA at site D in the nucleolus, since Nob1 is associated with pre-ribosomes and the 20S pre-rRNA and it is present in both the nucleus and cytoplasm^5^,^8^,^9^.

We propose that Nop9 protects ITS1 from premature cleavage in the nucleolus by blocking access of Nob1 to its binding site near site D and therefore inhibiting substrate recognition and cleavage. Nop9 appears to join the assembling SSU processome/90S pre-ribosome before Nob1, which is consistent with this inhibitory role^9^. Nop9 may first associate with the pre-ribosome via protein–protein interactions or through another RNA site, since it associates with pre-rRNA fragments lacking ITS1. It may also participate in regulating early processing of the 35S pre-rRNA at the A0, A1 and A2 sites^11^,^12^. Nop9 would not prevent association of Nob1 with the 20S pre-rRNA in the nucleolus, as Nob1 binds to recognition sites located upstream of site D (nucleotides −46 to −34) as well as to other sites within ITS1 (refs 6, 23). Nop9 dissociates from the pre-ribosome before or during export to the cytoplasm, as green fluorescent protein-tagged Nop9 is nucleolar and not associated with cytoplasmic pre-40S particles^7^,^11^,^26^. When Nop9 leaves the pre-ribosome, the region near site D of 20S pre-rRNA becomes accessible for Nob1 recognition and cleavage. Additional mechanisms then control maturation to 18S rRNA in cytoplasmic pre-40S particles and assure that translationally competent pre-40S ribosomes associate with mature 60S ribosomal subunits. For example, cytoplasmic pre-40S assembly factors prevent premature association of translation initiation factors and regulate pre-rRNA structural changes, and site D cleavage requires ATP binding by Rio1 (refs 7, 8, 23, 27, 28, 29). Thus, Nop9's role in controlling site D cleavage in the nucleolus is an important part of the orchestrated process of pre-rRNA maturation progressing through different cellular compartments.

**Figure 7:**
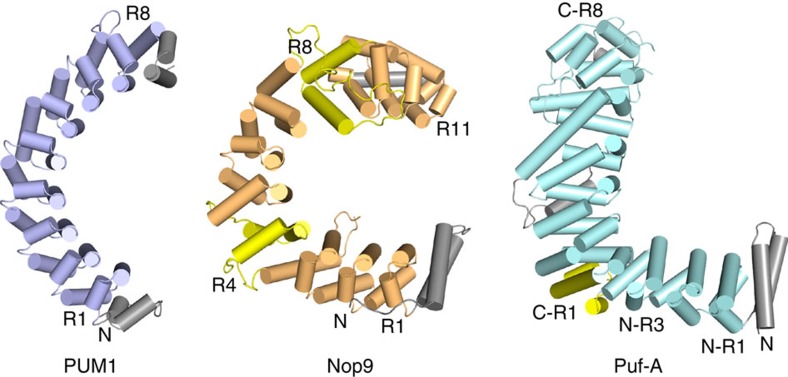
PUM repeats form three distinct classes of RNA-binding proteins. Schematic drawings of the crystal structures of human PUM1 (left), yeast Nop9 (centre) and human Puf-A (right). PUM repeats at structural junctions in Nop9 and Puf-A are coloured yellow, and pseudorepeats at the N- and C termini are coloured grey. Puf-A comprises 11 PUM repeats, which are numbered N-R1 to N-R3 and C-R1 to C-R8 to indicate that its C-terminal repeats C-R1 to C-R8 form a curved structure similar to PUM1 (ref. 21).

PUM repeat-containing proteins fall into three classes based on sequence homology: (1) the classical PUM/FBF family, (2) the Puf-A/Puf6 family and (3) the Nop9 family. With this study, we establish that the three classes display different structures with distinct RNA-recognition properties (Fig. 7). Classical crescent-shaped PUF proteins contain eight PUM repeats and recognize single-stranded RNA through sequence-specific contacts with the RNA bases^16^,^17^. Puf-A/Puf6 family proteins, which are L-shaped, contain 11 PUM repeats and interact with single- or double-stranded RNA through non-sequence-specific contacts with the phosphate backbone^21^. Puf-A/Puf6 proteins use residues from the α2 helices to recognize double-stranded RNA; however, the RNA interaction motifs are divergent. The study here reveals that Nop9's 11 PUM repeats form a C shape and interact with an ITS1 pre-rRNA target comprising 5′ and 3′ single-stranded regions flanking a duplex region. Nop9 recognizes both single-stranded and duplex elements, and both sequence and structure appear to be important for high-affinity binding. All PUM repeat-containing proteins may utilize their distinct recognition properties to function in mRNA regulation, as NOP9/c14orf21 and Puf-A/KIAA0020 have been identified as mRNA-binding proteins in mammals^30^,^31^. Protein:protein interactions are also critical to establish effector complexes on RNA targets, and it is likely that Puf-A/Puf6 and Nop9 proteins, like classical PUF proteins, form complexes with other proteins. Nop9 may associate with the SSU processome/90S pre-ribosome through protein–protein interactions before its 20S pre-rRNA target site is transcribed^9^. Identification of these interactions will facilitate the discovery of mechanisms in ribosome assembly or additional cellular processes.

Our SAXS model of the Nop9:RNA complex in conjunction with biochemical probing suggests that Nop9 recognizes specific sequence and structural elements within ITS1 of the pre-rRNA. The N-terminal residues of Nop9 preceding repeat R1′ (residues 50–58) are packed along the α1 helices of repeats R1 and R2, and the sequence is well conserved. The SAXS model suggests that this peptide might interact with the major groove of the double-stranded region of ITS1. In addition, interaction of the N-terminal repeats of Nop9 with the duplex region of the RNA includes residues at the junction between the α1 and α2 helices of repeats R1–R3. This interaction may be similar to minor groove interaction by equivalent residues of Puf-A^21^, suggesting a conserved feature. The SAXS model cannot resolve single-stranded RNA-recognition features of Nop9. SϕxxE/D motifs are the most frequently occurring RNA-recognition motif on the concave surface of Nop9. In the context of classical PUF proteins, the SϕxxE/D motif would recognize G (for example, repeat 7 of *Caenorhabditis elegans* FBF-2)^32^. However, the only guanine found in the Nop9-binding site in ITS1 is G19, base-paired with C204, and mutation to cytosine (G19C/C204G) did not affect binding affinity. In addition, NMR analyses suggested that short G-rich RNAs show only very weak binding to Nop9 (Supplementary Fig. 8). Surprisingly, Nop9 binds to short A-rich RNAs, as shown by saturation-transfer difference NMR (Supplementary Fig. 8d). Therefore, the SϕxxE/D motifs in Nop9 may have different recognition properties than selecting guanine bases, as in classical PUF proteins and as selected by the plant Nop9 homologue, APUM23 (refs 17, 33). A crystal structure of Nop9 in complex with ITS1 RNA will aid in deciphering its base-specific recognition code.

The extended PUF family of proteins has achieved a remarkable degree of functional diversity for its small size. Most eukaryotic organisms possess a set of classical PUF proteins that regulate mRNA translation of specific targets, one Puf-A/Puf6 protein involved in large ribosomal subunit biogenesis and one Nop9 protein involved in small ribosomal subunit biogenesis. Our results here suggest that Nop9 may represent an evolutionary bridge between the sequence-specific classical PUF proteins and sequence-independent Puf-A/Puf6 proteins (Fig. 7). Nop9's 11 PUM repeats form a C shape, extending the classical PUF protein curvature, but with twists focused at some repeats. As the protein family evolved, introducing twists might have buried some classical PUM repeat RNA-recognition residues, thus diminishing sequence-specific recognition. *Arabidopsis thaliana* APUM23, a Nop9 family member, appears to have evolved distinct RNA sequence recognition properties from yeast Nop9, further diversifying this group of proteins^33^. The Puf-A/Puf6 proteins exaggerate a twist to produce a joint and a distinctive L shape. This small family of proteins provides an example of natural engineering of curvature change to evolve distinct ligand specificity. Understanding PUF protein curvature changes may serve as a template to guide design of synthetic proteins comprising α-helical repeats to recognize specific ligands as well as presenting the opportunity to engineer PUF RNA-binding proteins that recognize structural as well as sequence features^34^,^35^.

## Methods

### Nop9 expression and purification

A cDNA fragment encoding full-length Nop9 (residues 1–666) was amplified from *S. cerevisiae* genomic DNA and cloned into pSMT3 (Memorial Sloan Kettering Cancer Center) using *Sac*I and *Not*I restriction sites. A cDNA fragment encoding a truncated protein (residues 46–645) was cloned similarly. Nop9 proteins were expressed at 22 °C overnight in *Escherichia coli* strain BL21-CodonPlus (DE3) in the presence of 0.4 mM isopropyl-β-D-thiogalactoside (IPTG), which was added when the OD_600_ reached 0.6. Cell pellets were resuspended in sonication buffer (25 mM HEPES, pH 7.5, 1 M NaCl, 1 mM TCEP [tris(2-carboxyethyl)phosphine], 25 mM imidazole) plus 1 mg ml^−1^ lysozyme and lysed by sonication, followed by centrifugation to remove cell debris. The supernatant was applied to 5 ml of HisPur Ni-NTA resin (Thermo Scientific), washed with 200 ml of sonication buffer and eluted with 25 mM HEPES, pH 7.5, 500 mM NaCl, 1 mM TCEP and 500 mM imidazole. The N-terminal SUMO-tagged protein was cleaved overnight with 2 μg ml^−1^ of Ulp1 at 4 °C. The cleaved sample was diluted into 20 mM HEPES, pH 7.5, 20 mM NaCl and 1 mM TCEP, and then it was loaded on a 5-ml HiTrap Heparin column (GE Healthcare). The sample was eluted with a linear gradient from 0 to 2 M NaCl in 20 mM HEPES and 1 mM TCEP. Nop9 eluted from the heparin column when the salt concentration reached ∼600 mM NaCl and was further purified using a HiLoad 16/60 Superdex200 column (GE Healthcare) equilibrated with 25 mM HEPES, pH 7.5, 500 mM NaCl and 1 mM TCEP. Truncated proteins were expressed and purified with the same protocol. Uncleaved SUMO–Nop9 or SUMO was purified using the same protocol with minor modifications: the Ulp1 cleavage step was omitted and SUMO protein was eluted from the heparin column when the salt concentration reached 300 mM NaCl. The identities of the proteins were confirmed using mass spectrometry, and the purities were >95% based on SDS–PAGE. Selenomethionine (SeMet)-substituted Nop9 was prepared by growing cells in M9 medium supplemented with SeMet (50 mg l^−1^), Lys (100 mg l^−1^), Thr (100 mg l^−1^), Phe (100 mg l^−1^), Leu (50 mg l^−1^), Ile (50 mg l^−1^) and Val (50 mg l^−1^). SeMet-containing protein was purified with the same protocol as for native protein, except all buffers were degassed for 2 h. Incorporation of selenium was >97% according to mass spectrometry. Nucleotide sequences for all plasmids were confirmed using DNA sequencing.

### Nob1 expression and purification

Wild-type *S. cerevisiae* Nob1 and inactive Nob1 D15N mutant proteins were cloned, expressed and purified similarly to Nop9 with minor modifications. Briefly, a cDNA encoding Nob1 was cloned into pSMT3 using *Sac*I and *Not*I restriction sites. Inactive Nob1 D15N mutant was prepared by site-directed mutagenesis PCR. Nucleotide sequences for both plasmids were confirmed using DNA sequencing. Nob1 proteins were expressed at 22 °C overnight in *E. coli* strain BL21-CodonPlus (DE3) in the presence of 0.5 mM IPTG, which was added when the OD_600_ reached 0.6 and 1 mM ZnCl_2_^6^. Wild-type Nob1 and its D15N mutant were purified using the same buffers and columns as Nop9. Purified proteins were concentrated to 150 μM in a buffer containing 25 mM HEPES, pH 7.5, 500 mM NaCl and 1 mM TCEP. The wild-type and mutant Nob1 proteins were purified with the same batch of buffers and the same set of chromatography columns using the same fast protein liquid chromatography system to control for any possible nuclease contamination.

### Protein crystallization and structure determination

A truncated construct of Nop9 (residues 46–645) was expressed and purified for crystallization. Truncation did not affect RNA binding (full-length Nop9 binds to Subdomain A Δ5′_1–6_, Δ3′ with a *K*_d_ of 10.1±1.0 nM versus 11.8±0.4 nM for the truncated protein, ± indicates s.e.m., *n*=3, *P* value=0.14). Initial crystals were obtained by hanging drop vapour diffusion at 22 °C, mixing 2 μl of 10 mg ml^−1^ Nop9 with 2 μl of a crystallization solution containing 18% (*w*/*v*) PEG 3350 and 0.2 M ammonium citrate. Crystals were improved by iterative microseeding. Crystallization solution was supplemented with 7.5% (*v*/*v*) MPD as a cryoprotectant. SeMet-substituted Nop9 crystallized in the same space group with slight changes in unit cell dimensions (Table 1).

X-ray diffraction data were collected at beamline 22-ID of the Advanced Photon Source at 100 K with a wavelength of 1.000 Å for native crystals and 0.979 Å for SeMet derivatives. The data were processed using HKL 2000 (ref. 36). *R*_pim_ (0.046 overall and 0.303 for 2.17–2.09 Å) and CC_1/2_ (0.996 overall and 0.769 for 2.17–2.09 Å) were calculated for unmerged data using Phenix.merging_statistics^37^. A SeMet crystal diffracted to 2.64 Å, and the resolution limit of the anomalous signal was 3.2 Å. Phases were determined by single-wavelength anomalous dispersion experiments using Phenix AutoSol, followed by AutoBuild to generate the initial model. With the initial single-wavelength anomalous dispersion model and the 2.1 Å native data, iterative refinement and model building using Phenix.Refine^37^ and WinCoot^38^ yielded final *R* and free *R* factors of 20.1% and 23.6%, respectively. There are two molecules in an asymmetric unit, and 57 residues (45–49, 164v174, 220–250 and 635–645) are not modelled. Over 96% of dihedral angles are in favoured regions of the Ramachandran plot, and only 0.19% are Ramachandran plot outliers.

### RNA preparation

DNA templates for *in vitro* transcription of ITS1_D-A2 (nucleotides 1–212) and pre-rRNA corresponding to the region containing nucleotides −164 to 212 were prepared by PCR amplification from *S. cerevisiae* genomic DNA (Supplementary Table 5) and purified with 1.2% agarose gel. DNA templates for ITS1 Subdomains A (nucleotides 1–38_184–212), B (nucleotides 39–77_141–183) and C (nucleotides 77–140) and ITS1 RNA mutants were purchased from Eurofins MWG Operon (Supplementary Table 5) and purified by denaturing PAGE gel. The T7 promoter sequence (5′-GAAATTAATACGACTCACTATA-3′) was used for *in vitro* transcription of RNA using T7 RNA polymerase, and two guanine bases were included to promote transcription. *In vitro* transcription samples were incubated at 37 °C overnight in 100 mM Tris-HCl, pH 8.0, 20 mM MgCl_2_, 1 mM TCEP, 2 mM spermidine, 3% (w/v) PEG 8000, 0.01% (*v*/*v*) Triton X-100, 4 mM nucleotide triphosphates (NTPs), 2 units of inorganic pyrophosphatase, 0.6 μM double-stranded DNA template and 0.06 mg ml^−1^ T7 RNA polymerase. *In vitro* transcription samples were treated with 10 units of alkaline phosphatase (New England Biolabs) at 37 °C for 30 min and purified by 15% polyacrylamide gel (30 cm × 40 cm × 1.6 mm) in the presence of 8 M urea and 1 × TBE. The gels were pre-run for 1 h to a temperature of 50 °C, and RNA samples were resolved under a constant power of 80 watts for 10 h, during which RNAs travel about three quarters of the gel length. The excised slices of gel containing target RNAs were dialysed overnight in 10 mM Tris-HCl, pH 8, and 1 mM EDTA. The purified RNAs were incubated at 90 °C for 2 min and snap-cooled on ice to refold the RNA. The homogeneity of refolded RNA was confirmed by single bands on 10% polyacrylamide native TBE gels (Invitrogen). Short RNAs corresponding to ITS1 nucleotides −46 to −34 (5′-AAAGUCGUAACAA-3′), nucleotides 1–16 (5′-AAGAAAUUUAAUAAUU-3′) and nucleotides 201–212 (5′-UUUCAAUACAAC-3′) were purchased from Dharmacon (Thermo Scientific), de-protected according to the vendor's instructions and used without further purification. RNA secondary structures were predicted using Mfold^39^, and the graphics in Figs 2 and 3 were prepared using VARNA^40^. In order to assess sequence changes without disrupting overall RNA folding, we used a fragment lacking the 5′ nucleotides 1–6 and the 3′ single-stranded region, nucleotides 201–212, for mutational analysis.

### EMSAs

RNAs were labelled at the 5′ end with ^32^P-γ-ATP (PerkinElmer Life Science) with T4 polynucleotide kinase for 1 h at 37 °C. Unincorporated ^32^P-γ-ATP was removed using Illustra MicroSpin G-25 columns. Radiolabelled RNAs (<50 pM) were incubated with protein samples at 4 °C for 40 min in 10 mM HEPES, 150 mM NaCl, 0.01% (*v*/*v*) Tween-20, 0.1 mg ml^−1^ bovine serum albumin (BSA) and 1 mM TCEP. The samples were resolved on 10% polyacrylamide native TBE gels at constant voltage (100 V) with 1 × TBE buffer at 4 °C for 35 min. The gels were dried and exposed overnight to storage phosphor screens that were then scanned on a Molecular Dynamics Typhoon PhosphorImager. Band intensities were quantified with ImageQuant 5.2. The data were fit using the Hill equation with GraphPad Prism 6. EMSAs were performed at least three times, and the mean *K*_d_'s and s.e.m. are reported. We conducted triplicate technical replicates for EMSAs, a customary sample size that provides the power to detect statistically significant difference, if present.

### SAXS analysis

SAXS data (0.013 Å^−1^<*q*<0.328 Å^−1^) were collected at the SIBYLS beamline (12.3.1) of the Advanced Light Source at room temperature. Nop9 (46–645) was exchanged into 25 mM HEPES, pH 7.5, 500 mM NaCl and 2 mM dithiothreitol (DTT) using size exclusion chromatography. ITS1 RNA (nucleotides 7–38_184–206) was dialysed into 25 mM HEPES, pH 7.5, 100 mM NaCl and 2 mM DTT. To purify the Nop9:ITS1 RNA complex, RNA and Nop9 were mixed at a 2:1 molar ratio and unbound RNA was removed by a Superdex 75 10/300 GL column (GE Healthcare) equilibrated in 25 mM HEPES, pH 7.5, 250 mM NaCl and 2 mM DTT. All samples were prepared at three concentrations (Supplementary Table 4), and the corresponding chromatography buffer or dialysate was used as SAXS reference.

SAXS data were analysed with the ATSAS package (2.5.2; ref. 41). Guinier analysis was carried out using PRIMUS to determine the radius of gyration (*R*_g_). An ITS1 RNA model (nucleotides 7–38_184–206) was predicted using the RNAComposer server^42^. The crystal structure of Nop9 lacked 57 loop residues (46–49, 164–174, 220–250 and 635–645) that could not be modelled. Cα atoms were built using the EOM package of ATSAS. We found that inclusion of the loop Cα atoms built into arbitrary conformations improved SAXS data fitting for the model of Nop9 protein alone. Rigid body modelling using the crystal structure of Nop9 and the predicted structure of ITS1 RNA as input models to fit the SAXS data of the Nop9:ITS1 RNA complex was carried out with SASREF^43^. The 5′ single-stranded region of ITS1 (nucleotides 7–16) was not included in the rigid body modelling, since the lack of conformational adjustment for this flexible region may make it a steric hindrance in rigid body modelling. Constraints were included to place the artificial loop-bridging RNA nucleotides 38 and 184 away from Nop9, since truncation of the extended RNA stem loop did not affect the Nop9:ITS1 RNA interaction. For all models, *χ*^2^-values for the fit of the experimental model to the corresponding SAXS data were calculated using the FoXS web server that optimizes hydration layer, and excluded volume and implicit hydrogens^44^,^45^ (Supplementary Fig. 4).

### Yeast analysis

*GAL::3HA-NOP9*, *GAL::3HA-NOB1* and *GAL::3HA-NOP9,3HA-NOB1* strains carrying a chromosomally integrated galactose-inducible/glucose-repressible promoter with a 3 × HA epitope tag at the N terminus of the protein product of *NOP9* and/or *NOB1* were created in the parent strain YPH499 (*MAT a ura3-52 lys2-80 ade2-101 trp1-Δ63 his3-Δ200 leu2-Δ1)*^46^ using the plasmids pFA6a-kanMX6-PGAL1-3xHA (*GAL::3HA-NOP9* and *GAL::3HA-NOB1*) or pFA6a-His3MX6-PGAL1-3xHA (*GAL::3HA-NOP9* (Kan); *GAL::3HA-NOB1* (His)) and oligonucleotide primers complementary to *NOP9* or *NOB1* (ref. 47); Supplementary Table 5). To assess the effect of depletion of Nop9 and/or Nob1 expression, parent YPH499, *GAL::3HA-NOP9*, *GAL::3HA-NOB1* and *GAL::3HA-NOP9; GAL::3HA-NOB1* yeast strains were first grown (YPG/R; 1% (*w*/*v*) yeast extract, 2% (*w*/*v*) peptone, 2% (*w*/*v*) galactose and 2% (*w*/*v*) raffinose) to early log phase at 30 °C and then shifted to YPD (1% (*w*/*v*) yeast extract, 2% (*w*/*v*) peptone and 2% (*w*/*v*) glucose (dextrose)) for 24 h to repress expression of endogenous Nop9 and/or Nob1. The cells were maintained in mid-log phase (OD_600_<0.8) by dilution of the culture with fresh YPD medium. Growth was monitored by OD_600_ measurement for 24 h. Depletion of endogenous Nop9 and/or Nob1 was confirmed with anti-HA (Abcam, catalogue number ab9110, 1:20,000 dilution) and anti-MPP10 (1:5,000 dilution^48^) western blot (Supplementary Fig. 5b). RNA was harvested and analysed by northern blot^49^ using oligonucleotide probes complementary to either the 18S rRNA (oligo a) or to the 25S rRNA (oligo y) to ITS1 between sites D and A2 (oligo b), or to ITS1 between sites A2 and A3 (oligo c; Fig. 1a and Supplementary Table 5). As a loading control, a probe complementary to the Scr1 RNA (oligo Scr1) was used. The mature and pre-rRNAs were quantified on a Molecular Dynamics Typhoon 8600 Variable Mode Imager. Representative full northern blots of the portions shown in Fig. 5c are presented in Supplementary Fig. 5b. We conducted triplicate biological replicates, a customary sample size that provides the power to detect statistically significant differences, if present.

The Nop9 cDNA was recombinantly cloned (Gateway, Invitrogen) into a modified version of the yeast expression vector p414GPD-3xFLAG-GW (ref. 21). The nucleotide sequence was confirmed using DNA sequencing. For depletion experiments performed at 17 °C, the *GAL::3HA-NOP9* yeast strain was transformed with the empty vector (−Nop9) or Nop9 (+Nop9) in p414GPD-3xFLAG. To assay growth in liquid medium, yeast strains were first grown in medium containing 2% (*w*/*v*) galactose and 2% (*w*/*v*) raffinose and lacking tryptophan (SG/R-Trp) to early log phase at 30 °C and then shifted to medium containing 2% (*w*/*v*) glucose (dextrose) and lacking tryptophan (SD-Trp) at 17 °C for 72 h to repress expression of endogenous Nop9. The cells were maintained in the mid-log phase (OD_600_<0.8) by dilution of the culture with fresh SD-Trp medium. Growth was monitored by OD_600_ measurement for 72 h. RNA was harvested 72 h after the shift and analysed on a northern blot using oligonucleotide probes complementary to either the 18S rRNA (oligo a) or to the 25S rRNA (oligo y), to ITS1 between site D and site A2 (oligo b), or to ITS1 between sites A2 and A3 (oligo 003; Fig. 1a and Supplementary Table 5). As a loading control, a probe complementary to the Scr1 RNA (oligo Scr1) was used. The mature and pre-rRNAs were quantified on a Bio-Rad Personal Molecular Imager. We conducted triplicate biological replicates, a customary sample size that provides the power to detect statistically significant differences, if present.

### *In vitro* Nob1 cleavage assays

Nob1 cleavage assays were carried out as described previously^8^ with minor modifications. Briefly, in a 20-μl reaction system, 3.5 μM Nob1, with or without Nop9, SUMO–Nop9 (0.5 or 1 μM) or SUMO (1 μM), was pre-incubated at 30 °C for 5 min in 25 mM HEPES, pH 7.5, 75 mM NaCl, 2 mM DDT, 0.1 mg ml^−1^ BSA, 0.8 unit μl^−1^ RNasin (Promega), 4.5% (*v*/*v*) glycerol, 0.3 μM yeast tRNA and 5 mM MnCl_2_, before addition of 1 μM pre-rRNA substrate including 164 nucleotides from the 3′ end of 18S rRNA and the nucleotides in ITS1 from sites D to A2 (nucleotides −164 to 212). Cleavage reactions were incubated for 10, 30 and 60 min, followed by proteinase K treatment at 37 °C for 90 min. After phenol/chloroform extraction and ethanol precipitation, the samples were dissolved in 10 μl TE buffer. Typically, 2 μl of cleavage reaction were mixed with 1 μl 0.5 μM ^32^P-labelled DNA primer (ITS1 nucleotides 62–84, Supplementary Table 5), incubated at 65 °C for 5 min and then placed immediately on ice. Primer extension was carried out at 57 °C for 20 min with 1 × Superscript IV buffer (Invitrogen), 2.5 mM DTT, 0.5 mM of each dNTP, 0.8 unit μl^−1^ RNasin (Promega) and 20 units of Superscript IV reverse transcriptase (Invitrogen). For sequencing lanes, reactions were supplemented with 0.4 mM of the respective ddNTP. At the end of primer extension, RNA templates were removed by incubating the samples with 50 mM NaOH at 95 °C for 2 min. Samples were neutralized with 50 mM HCl, 300 mM sodium acetate and ethanol precipitated. Two-microlitre samples dissolved in formamide loading buffer were resolved on 8% polyacrylamide-denaturing gels (31.0 cm × 38.5 cm × 0.4 mm) at a constant power of 65 W for 150 min. The dried gels were exposed to storage phosphor screens for 2 h and scanned using a Molecular Dynamics Typhoon PhosphorImager. Band intensities were quantified with ImageQuant 5.2. Cleavage profiles were generated using SAFA^50^. The full gel of the portion shown in Fig. 6a is presented in Supplementary Fig. 7a. We conducted triplicate technical replicates, a customary sample size that provides the power to detect statistically significant differences, if present.

### Saturation-transfer difference NMR

Short RNA (1 mM), 5′-CGG-3′ or 5′-CAA-3′(Dharmacon, Thermo Scientific), was mixed with 40 μM Nop9 (46–645) in D_2_O buffer containing 50 mM sodium phosphate pH 7.2, 200 mM NaCl and 1 mM TCEP. STD-NMR data were collected on a 600 MHz magnet at 298 K using the reported pulse sequence^51^. Overall, 4,096 transients were accumulated for all experiments.

### Data availability

Coordinates and structure factors have been deposited in the Protein Data Bank with accession ID 5SVD. The authors declare that all other data supporting the findings of this study are available within the article and its Supplementary Information files or from the corresponding author upon request.

## Additional information

**How to cite this article:** Zhang, J. *et al*. Nop9 is a PUF-like protein that prevents premature cleavage to correctly process pre-18S rRNA. *Nat. Commun.* **7**, 13085 doi: 10.1038/ncomms13085 (2016).

## Supplementary Material

Supplementary Information Supplementary Figures 1 - 8, Supplementary Table 1 - 5 and Supplementary References

## Figures and Tables

**Table 1:** Data collection and refinement statistics.

	**SeMet Nop9 (46**–**645)**	**Nop9 (46**–**645)**
*Data collection*		
Space group	P2_1_	P2_1_
Cell dimensions		
*a*, *b*, *c* (Å)	67.58, 109.14, 115.04	67.50, 110.47, 114.85
β (°)	93.3	92.2
Resolution (Å)	50–2.65 (2.7–2.65)*	50–2.1 (2.14–2.1)
*R*_sym_	0.114 (0.54)	0.086 (0.43)
*I*/σ*I*	14.3 (2.75)	12.5 (3.34)
Completeness (%)	96.4 (72.0)	97.5 (86.4)
Redundancy	13.2 (6.8)	7.0 (4.0)
*Refinement*		
Resolution (Å)		32.0–2.1
No. of reflections		96,076
*R*_work_/*R*_free_		18.5%/22.6%
No. of atoms		9,186
Protein		8,809
Water/ion		377
*B*-factors		
Protein		58.4
Water/ion		53.7
R.m.s. deviations		
Bond lengths (Å)		0.009
Bond angles (°)		1.11

SeMet, selenomethionine.

^*^Values in parentheses are for the highest-resolution shell.

## References

[b1] de la Cruz J., Karbstein K. & Woolford J. L. Jr Functions of ribosomal proteins in assembly of eukaryotic ribosomes *in vivo*. Annu. Rev. Biochem. 84, 93–129 (2015). 25706898 10.1146/annurev-biochem-060614-033917 PMC4772166

[b2] Henras A. K., Plisson-Chastang C., O'Donohue M. F., Chakraborty A. & Gleizes P. E. An overview of pre-ribosomal RNA processing in eukaryotes. Wiley Interdiscip. Rev. RNA 6, 225–242 (2015). 25346433 10.1002/wrna.1269 PMC4361047

[b3] Lafontaine D. L. Noncoding RNAs in eukaryotic ribosome biogenesis and function. Nat. Struct. Mol. Biol. 22, 11–19 (2015). 25565028 10.1038/nsmb.2939

[b4] Woolford J. L. Jr & Baserga S. J. Ribosome biogenesis in the yeast *Saccharomyces cerevisiae*. Genetics 195, 643–681 (2013). 24190922 10.1534/genetics.113.153197 PMC3813855

[b5] Fatica A., Oeffinger M., Dlakic M. & Tollervey D. Nob1p is required for cleavage of the 3′ end of 18S rRNA. Mol. Cell Biol. 23, 1798–1807 (2003). 12588997 10.1128/MCB.23.5.1798-1807.2003 PMC151717

[b6] Lamanna A. C. & Karbstein K. Nob1 binds the single-stranded cleavage site D at the 3′-end of 18S rRNA with its PIN domain. Proc. Natl Acad. Sci. USA 106, 14259–14264 (2009). 19706509 10.1073/pnas.0905403106 PMC2732849

[b7] Strunk B. S. . Ribosome assembly factors prevent premature translation initiation by 40S assembly intermediates. Science 333, 1449–1453 (2011). 21835981 10.1126/science.1208245 PMC3402165

[b8] Lebaron S. . Proofreading of pre-40S ribosome maturation by a translation initiation factor and 60S subunits. Nat. Struct. Mol. Biol. 19, 744–753 (2012). 22751017 10.1038/nsmb.2308 PMC3654374

[b9] Chaker-Margot M., Hunziker M., Barandun J., Dill B. D. & Klinge S. Stage-specific assembly events of the 6-MDa small-subunit processome initiate eukaryotic ribosome biogenesis. Nat. Struct. Mol. Biol. 22, 920–923 (2015). 26479197 10.1038/nsmb.3111

[b10] Pertschy B. . RNA helicase Prp43 and its co-factor Pfa1 promote 20 to 18S rRNA processing catalyzed by the endonuclease Nob1. J. Biol. Chem. 284, 35079–35091 (2009). 19801658 10.1074/jbc.M109.040774 PMC2787369

[b11] Thomson E., Rappsilber J. & Tollervey D. Nop9 is an RNA binding protein present in pre-40S ribosomes and required for 18S rRNA synthesis in yeast. RNA 13, 2165–2174 (2007). 17956976 10.1261/rna.747607 PMC2080597

[b12] Li Z. . Rational extension of the ribosome biogenesis pathway using network-guided genetics. PLoS Biol. 7, e1000213 (2009). 19806183 10.1371/journal.pbio.1000213 PMC2749941

[b13] Zhang L., Wu C., Cai G., Chen S. & Ye K. Stepwise and dynamic assembly of the earliest precursors of small ribosomal subunits in yeast. Genes Dev. 30, 718–732 (2016). 26980190 10.1101/gad.274688.115 PMC4803056

[b14] Nudel R. . Genome-wide association analyses of child genotype effects and parent-of-origin effects in specific language impairment. Genes Brain Behav. 13, 418–429 (2014). 24571439 10.1111/gbb.12127 PMC4114547

[b15] Edwards T. A., Pyle S. E., Wharton R. P. & Aggarwal A. K. Structure of Pumilio reveals similarity between RNA and peptide binding motifs. Cell 105, 281–289 (2001). 11336677 10.1016/s0092-8674(01)00318-x

[b16] Wang X., Zamore P. D. & Hall T. M. T. Crystal structure of a Pumilio homology domain. Mol. Cell 7, 855–865 (2001). 11336708 10.1016/s1097-2765(01)00229-5

[b17] Wang X., McLachlan J., Zamore P. D. & Hall T. M. T. Modular recognition of RNA by a human pumilio-homology domain. Cell 110, 501–512 (2002). 12202039 10.1016/s0092-8674(02)00873-5

[b18] Miller M. A. & Olivas W. M. Roles of Puf proteins in mRNA degradation and translation. Wiley Interdiscip. Rev. RNA 2, 471–492 (2011). 21957038 10.1002/wrna.69

[b19] Wharton R. P. & Aggarwal A. K. mRNA regulation by Puf domain proteins. Sci. STK. 2006, pe37 (2006). 10.1126/stke.3542006pe37 17003467

[b20] Wickens M., Bernstein D. S., Kimble J. & Parker R. A PUF family portrait: 3′UTR regulation as a way of life. Trends Genet. 18, 150–157 (2002). 11858839 10.1016/s0168-9525(01)02616-6

[b21] Qiu C., McCann K. L., Wine R. N., Baserga S. J. & Hall T. M. T. A divergent Pumilio repeat protein family for pre-rRNA processing and mRNA localization. Proc. Natl Acad. Sci. USA 111, 18554–18559 (2014). 25512524 10.1073/pnas.1407634112 PMC4284587

[b22] Cheong C. G. & Hall T. M. T. Engineering RNA sequence specificity of Pumilio repeats. Proc. Natl Acad. Sci. USA 103, 13635–13639 (2006). 16954190 10.1073/pnas.0606294103 PMC1564246

[b23] Turowski T. W. . Rio1 mediates ATP-dependent final maturation of 40S ribosomal subunits. Nucleic Acids Res. 42, 12189–12199 (2014). 25294836 10.1093/nar/gku878 PMC4231747

[b24] Guthrie C., Nashimoto H. & Nomura M. Structure and function of *E. coli* ribosomes. 8. Cold-sensitive mutants defective in ribosome assembly. Proc. Natl Acad. Sci. USA 63, 384–391 (1969). 4895536 10.1073/pnas.63.2.384 PMC223576

[b25] Noble S. M. & Guthrie C. Identification of novel genes required for yeast pre-mRNA splicing by means of cold-sensitive mutations. Genetics 143, 67–80 (1996). 8722763 10.1093/genetics/143.1.67 PMC1207296

[b26] Schafer T., Strauss D., Petfalski E., Tollervey D. & Hurt E. The path from nucleolar 90S to cytoplasmic 40S pre-ribosomes. EMBO J. 22, 1370–1380 (2003). 12628929 10.1093/emboj/cdg121 PMC151049

[b27] Garcia-Gomez J. J. . Final pre-40S maturation depends on the functional integrity of the 60S subunit ribosomal protein L3. PLoS Genet. 10, e1004205 (2014). 24603549 10.1371/journal.pgen.1004205 PMC3945201

[b28] Strunk B. S., Novak M. N., Young C. L. & Karbstein K. A translation-like cycle is a quality control checkpoint for maturing 40S ribosome subunits. Cell 150, 111–121 (2012). 22770215 10.1016/j.cell.2012.04.044 PMC3615461

[b29] Hector R. D. . Snapshots of pre-rRNA structural flexibility reveal eukaryotic 40S assembly dynamics at nucleotide resolution. Nucleic Acids Res. 42, 12138–12154 (2014). 25200078 10.1093/nar/gku815 PMC4231735

[b30] Baltz A. G. . The mRNA-bound proteome and its global occupancy profile on protein-coding transcripts. Mol. Cell 46, 674–690 (2012). 22681889 10.1016/j.molcel.2012.05.021

[b31] Castello A. . Insights into RNA biology from an atlas of mammalian mRNA-binding proteins. Cell 149, 1393–1406 (2012). 22658674 10.1016/j.cell.2012.04.031

[b32] Wang Y., Opperman L., Wickens M. & Hall T. M. Structural basis for specific recognition of multiple mRNA targets by a PUF regulatory protein. Proc. Natl Acad. Sci. USA 106, 20186–20191 (2009). 19901328 10.1073/pnas.0812076106 PMC2787170

[b33] Zhang C. & Muench D. G. A nucleolar PUF RNA-binding protein with specificity for a unique RNA sequence. J. Biol. Chem. 290, 30108–30118 (2015). 26487722 10.1074/jbc.M115.691675 PMC4706012

[b34] Javadi Y. & Itzhaki L. S. Tandem-repeat proteins: regularity plus modularity equals design-ability. Curr. Opin. Struct. Biol. 23, 622–631 (2013). 23831287 10.1016/j.sbi.2013.06.011

[b35] Park K. . Control of repeat-protein curvature by computational protein design. Nat. Struct. Mol. Biol. 22, 167–174 (2015). 25580576 10.1038/nsmb.2938 PMC4318719

[b36] Otwinowski Z. & Minor W. Processing of X-ray diffraction data collected in oscillation mode. Method Enzymol. 276, 307–326 (1997). 10.1016/S0076-6879(97)76066-X 27754618

[b37] Adams P. D. . PHENIX: a comprehensive Python-based system for macromolecular structure solution. Acta Crystallogr. D Biol. Crystallogr. 66, 213–221 (2010). 20124702 10.1107/S0907444909052925 PMC2815670

[b38] Emsley P., Lohkamp B., Scott W. G. & Cowtan K. Features and development of Coot. Acta Crystallogr. D Biol. Crystallogr. 66, 486–501 (2010). 20383002 10.1107/S0907444910007493 PMC2852313

[b39] Zuker M. Mfold web server for nucleic acid folding and hybridization prediction. Nucleic Acids Res. 31, 3406–3415 (2003). 12824337 10.1093/nar/gkg595 PMC169194

[b40] Darty K., Denise A. & Ponty Y. VARNA: interactive drawing and editing of the RNA secondary structure. Bioinformatics 25, 1974–1975 (2009). 19398448 10.1093/bioinformatics/btp250 PMC2712331

[b41] Petoukhov M. V., Konarev P. V., Kikhney A. G. & Svergun D. I. ATSAS 2.1—towards automated and websupported small-angle scattering data analysis. J. Appl. Cryst. 40, 6 (2007).

[b42] Popenda M. . Automated 3D structure composition for large RNAs. Nucleic Acids Res. 40, e112 (2012). 22539264 10.1093/nar/gks339 PMC3413140

[b43] Petoukhov M. V. & Svergun D. I. Global rigid body modeling of macromolecular complexes against small-angle scattering data. Biophys. J. 89, 1237–1250 (2005). 15923225 10.1529/biophysj.105.064154 PMC1366608

[b44] Schneidman-Duhovny D., Hammel M., Tainer J. A. & Sali A. Accurate SAXS profile computation and its assessment by contrast variation experiments. Biophys. J. 105, 962–974 (2013). 23972848 10.1016/j.bpj.2013.07.020 PMC3752106

[b45] Schneidman-Duhovny D., Hammel M. & Sali A. FoXS: a web server for rapid computation and fitting of SAXS profiles. Nucleic Acids Res. 38, W540–W544 (2010). 20507903 10.1093/nar/gkq461 PMC2896111

[b46] Sikorski R. S. & Hieter P. A system of shuttle vectors and yeast host strains designed for efficient manipulation of DNA in *Saccharomyces cerevisiae*. Genetics 122, 19–27 (1989). 2659436 10.1093/genetics/122.1.19 PMC1203683

[b47] Charette J. M. & Baserga S. J. The DEAD-box RNA helicase-like Utp25 is an SSU processome component. RNA 16, 2156–2169 (2010). 20884785 10.1261/rna.2359810 PMC2957055

[b48] Dunbar D. A., Wormsley S., Agentis T. M. & Baserga S. J. Mpp10p, a U3 small nucleolar ribonucleoprotein component required for pre-18S rRNA processing in yeast. Mol. Cell Biol. 17, 5803–5812 (1997). 9315638 10.1128/mcb.17.10.5803 PMC232428

[b49] Wehner K. A. & Baserga S. J. The sigma(70)-like motif: a eukaryotic RNA binding domain unique to a superfamily of proteins required for ribosome biogenesis. Mol. Cell 9, 329–339 (2002). 11864606 10.1016/s1097-2765(02)00438-0

[b50] Das R., Laederach A., Pearlman S. M., Herschlag D. & Altman R. B. SAFA: Semi-automated footprinting analysis software for high-throughput quantification of nucleic acid footprinting experiments. RNA 11, 344–354 (2005). 15701734 10.1261/rna.7214405 PMC1262685

[b51] Mayer M. & Meyer B. Group epitope mapping by saturation transfer difference NMR to identify segments of a ligand in direct contact with a protein receptor. J. Am. Chem. Soc. 123, 6108–6117 (2001). 11414845 10.1021/ja0100120

[b52] Schneider T. D. & Stephens R. M. Sequence logos: a new way to display consensus sequences. Nucleic Acids Res. 18, 6097–6100 (1990). 2172928 10.1093/nar/18.20.6097 PMC332411

[b53] Ashkenazy H., Erez E., Martz E., Pupko T. & Ben-Tal N. ConSurf 2010: calculating evolutionary conservation in sequence and structure of proteins and nucleic acids. Nucleic Acids Res. 38, W529–W533 (2010). 20478830 10.1093/nar/gkq399 PMC2896094

