# Supplementary material for: Nop9 is a PUF-like protein that prevents premature cleavage to correctly process pre-18S rRNA
Source: Nat Commun. 2016 Oct 11;7:13085. doi: 10.1038/ncomms13085 (PMC5062617; doi:10.1038/ncomms13085)
Supplement: Supplementary Information — Supplementary Figures 1 - 8, Supplementary Table 1 - 5 and Supplementary References [file ncomms13085-s1.pdf]

**Supplementary Information**

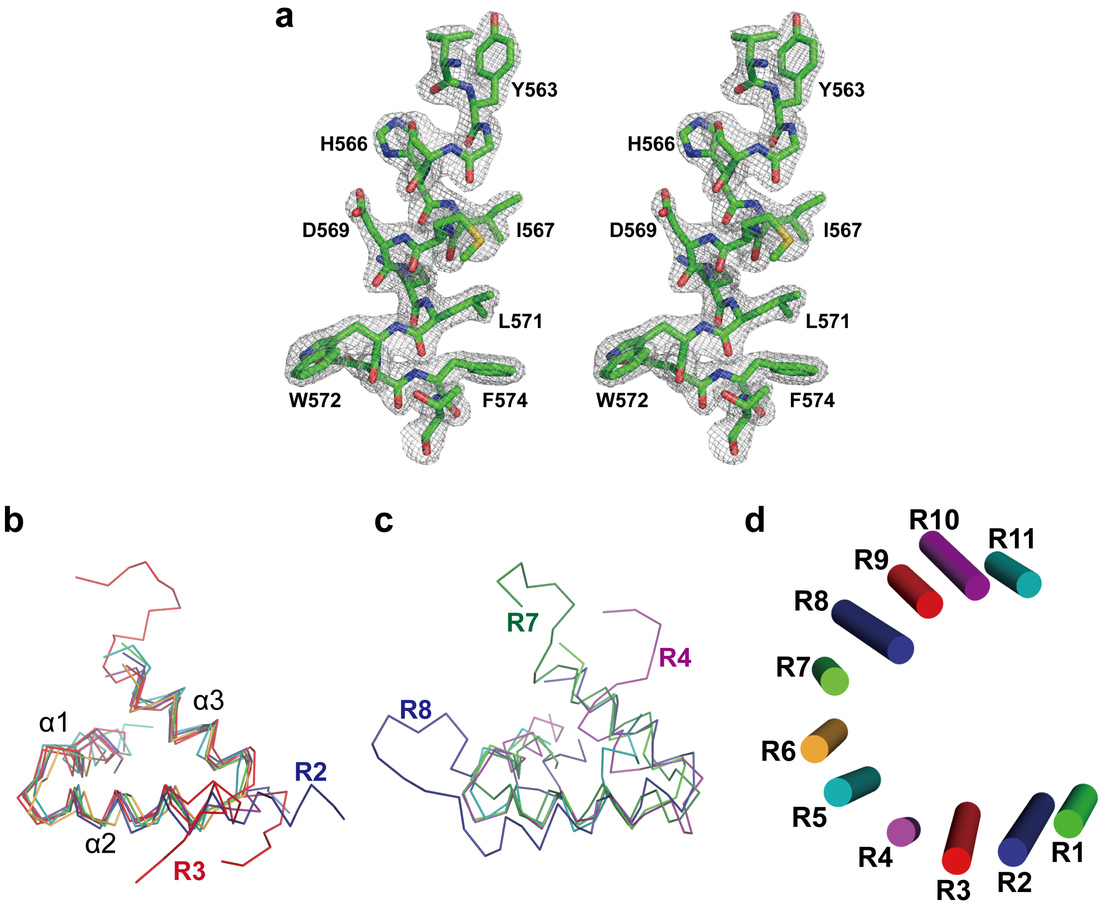


**Supplementary Figure 1.** Structure of the Nop9 PUM repeat protein family. (a) Stereo image of a representative portion of the Nop9 electron density map. Residues V562-T575 are shown superimposed with a $2F_o$-$F_c$ composite omit map contoured at 2.0 σ. (b) Superposition of Nop9 PUM repeats R1-R3, R5-R6, and R9-R10, which are structurally similar. (c) Superposition of Nop9 PUM repeats R4, R7, R8, and R11, which bear divergent loops that correspond to perturbations in repeat-to-repeat angles. (d) Nop9 PUM repeats form a twisted C shape. The α2 helices lining the concave surface of Nop9 are shown as cylinders to allow visualization of the major repeat-to-repeat twists focused around repeats R4 and R7. Repeats are colored as in Fig. 1b. In the absence of twisting, the curved inner surface of Nop9 could be rendered with the axes of all α2 helices orthogonal to the page.

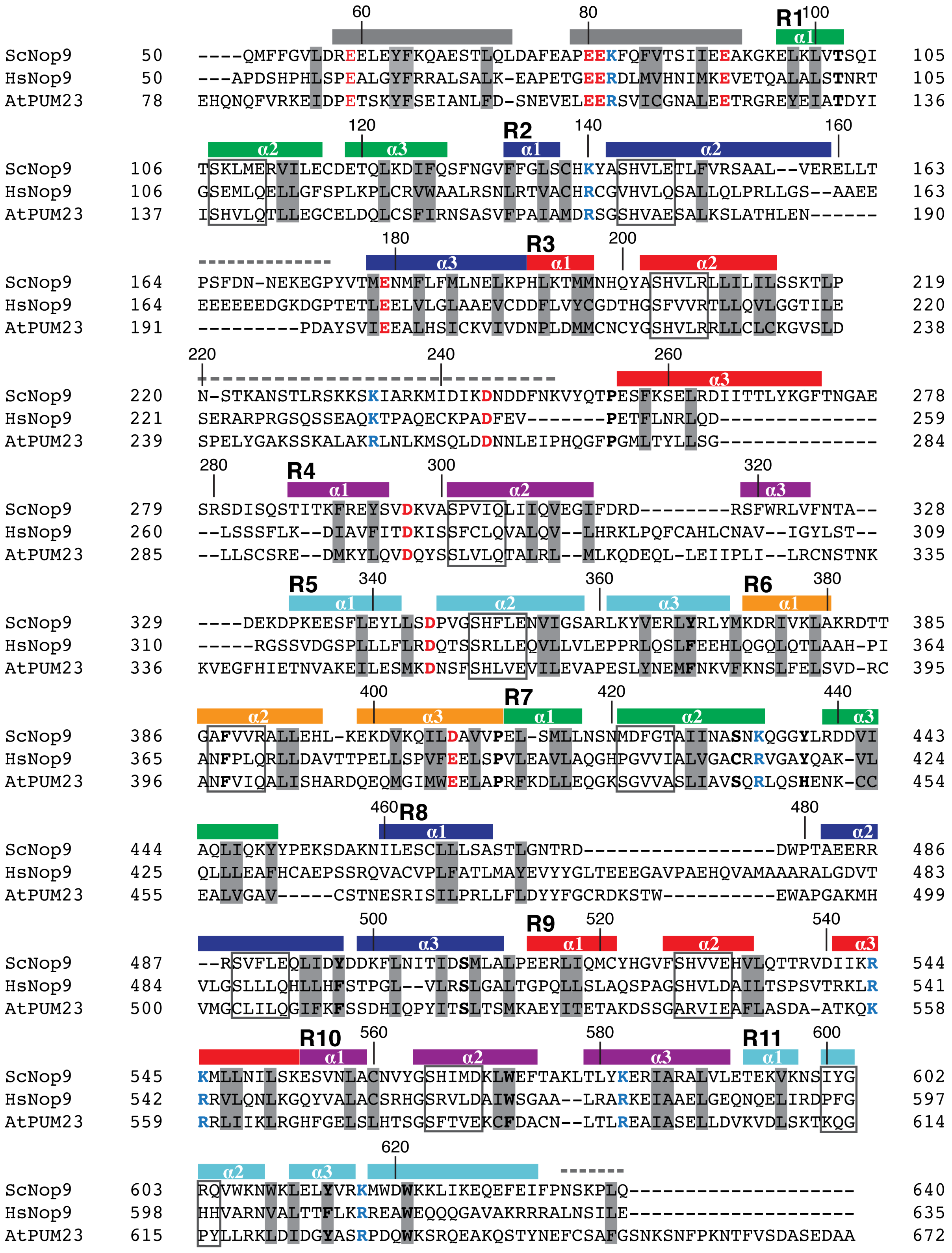
60 80 R1 100
α1
ScNop9 50 ----QMFFGVLDREELEYFKQAESTLQLDAFEAPEEKFQFVTSIIEEAKGKELKLVTSQI 105
HsNop9 50 ---APDSHPHLSPEALGYFRRALSALK-EAPETGEERDLMVHNIMKEVETQALALSTNRT 105
AtPUM23 78 EHQNQFVRKEIDPETSKYFSEIANLFD-SNEVELEERSVICGNALEETRGREYEIATDYI 136
120 R2 140 160
α2 α3 α1 α2
ScNop9 106 TSKLMERVILECDETQLKDIFQSFNGVFFGLSCHKYASHVLETLFVRSAAL--VERELLT 163
HsNop9 106 GSEMLQELLGFSPLKPLCRVWAALRSNLRTVACHRCGVHVLQSALLQLPRLLGS--AAEE 163
AtPUM23 137 ISHVLQTLLEGCELDQLCSFIRNSASVFPAIAMDRSGSHVAESALKSLATHLEN------ 190
180 R3 200
α3 α1 α2
ScNop9 164 PSFDN-NEKEGPYVTMENMFLFMLNELKPHLKTMMNHQYASHVLRLLILILSSKTLP 219
HsNop9 164 EEEEEEDGKDGPTETLEELVLGLAAEVCDDFLVYCGDTHGSFVVRTLLQVLGGTILE 220
AtPUM23 191 ---------PDAYSVIEEALHSICKVIVDNPLDMMCNCYGSHVLRRLLCLCKGVSLD 238
220 240 260
α3
ScNop9 220 N-STKANSTLRSKKSKIARKMIDIKDNDDFNKVYQTPESFKSELRDIITTLYKGFTNGAE 278
HsNop9 221 SERARPRGSQSSEAQKTPAQECKPADFEV-------PETFLNRLQD-------------- 259
AtPUM23 239 SPELYGAKSSKALAKRLNLKMSQLDDNNLEIPHQGFPGMLTYLLSG-------------- 284
280 R4 300 320
α1 α2 α3
ScNop9 279 SRSDISQSTITKFREYSVDKVASPVIQLIIQVEGIFDRD--------RSFWRLVFNTA-- 328
HsNop9 260 --LSSSFLK--DIAVFITDKISSFCLQVALQV--LHRKLPQFCAHLCNAV--IGYLST-- 309
AtPUM23 285 --LLSCSRE--DMKYLQVDQYSSLVLQTALRL--MLKQDEQL-LEIIPLI--LRCNSTNK 335
R5 340 360 R6 380
α1 α2 α3 α1
ScNop9 329 ----DEKDPKEESFLEYLLSDPVGSHFLENVIGSARLKYVERLYRLYMKDRIVKLAKRDTT 385
HsNop9 310 -----RGSSVDGSPLLLFLRDQTSSRLLEQVLLVLEPPRLQSLFEEHLQGQLQTLAAH-PI 364
AtPUM23 336 KVEGFHIETNVAKEILESMKDNSFSHLVEVILEVAPESLYNEMFNKVFKNSLFELSVD-RC 395
400 R7 420 440
α2 α3 α1 α2 α3
ScNop9 386 GAFVVRALLEHL-KEKDVKQILDAVVPEL-SMLLNSNMDFGTAIINASNKQGGYLRDDVI 443
HsNop9 365 ANFPLQRLLDAVTTPELLSPVFEELSPVLEAVLAQGHPGVVIALVGACRRVGAYQAK-VL 424
AtPUM23 396 ANFVIQALISHARDQEQMGIMWEELAPRFKDLLEQGKSGVVASLIAVSQRLQSHENK-CC 454
460 R8 480
α1 α2
ScNop9 444 AQLIQKYYPEKSDAKNILESCLLLSASTLGNTRD-----------------DWPTAEERR 486
HsNop9 425 QLLLEAFHCAEPSSRQVACVPLFATLMAYEVYYGLTEEEGAVPAEHQVAMAAARALGDVT 483
AtPUM23 455 EALVGAV-----CSTNESRISILPRLLFLDYYFGCRDKSTW----------EWAPGAKMH 499
500 R9 520 540
α3 α1 α2 α3
ScNop9 487 --RSVFLEQLIDYDDKFLNITIDSMLALPEERLIQMCYHGVFSHVVEHVLQTTRVDIIKR 544
HsNop9 484 VLGSLLLQHLLHFSTPGL--VLRSLGALTGPQLLSLAQSPAGSHVLDAILTSPSVTRKLR 541
AtPUM23 500 VMGCLILQGIFKFSSDHIQPYITSLTSMKAEYITETAKDSSGARVIEAFLASDA-ATKQK 558
R10 560 580 R11 600
α1 α2 α3 α1
ScNop9 545 KMLLNILSKESVNLACNVYGSHIMDKLWEFTAKLTLYKERIARALVLETEKVKNSIYG 602
HsNop9 542 RRVLQNLKGQYVALACSRHGSRVLDAIWSGAA--LRARKEIAAELGEQNQELIRDPFG 597
AtPUM23 559 RRLIIKLRGHFGELSLHTSGSFTVEKCFDACN--LTLREAIASELLDVKVDLSKTKQG 614
620
α2 α3
ScNop9 603 RQVWKNWKLELYVRKMWDWKKLIKEQEFEIFPNSKPLQ-------------------- 640
HsNop9 598 HHVARNVALTTFLKRREAWEQQQGAVAKRRRALNSILE--------------------- 635
AtPUM23 615 PYLLRKLDIDGYASRPDQWKSRQEAKQSTYNEFCSAFGSNKSNFPKNTFVSDASEDAA 672

**Supplementary Figure 2.** Protein sequence alignment of Nop9 family members. Amino acid sequences are shown for *S. cerevisiae* Nop9, human NOP9 and *Arabidopsis thaliana* APUM23. α helices are indicated by rectangles and colored by repeat as in Fig. 1b. Classical PUF protein RNA recognition motif sequences are boxed. Conserved hydrophobic residues are indicated with gray boxes, and conserved acidic and basic residues are colored red and blue, respectively. Residues disordered in the Nop9 crystal structure are indicated by dashed lines above the sequences. The sequences were aligned using ClustalX 2.1[1]. APUM23 is involved in 18S rRNA processing[2,3], but it is not an essential gene in *A. thaliana*. SELEX experiments identified a 10-nt RNA recognition sequence for APUM23, however, selection with a 20-nt randomized region did not identify a specific recognition sequence for yeast Nop9[4].

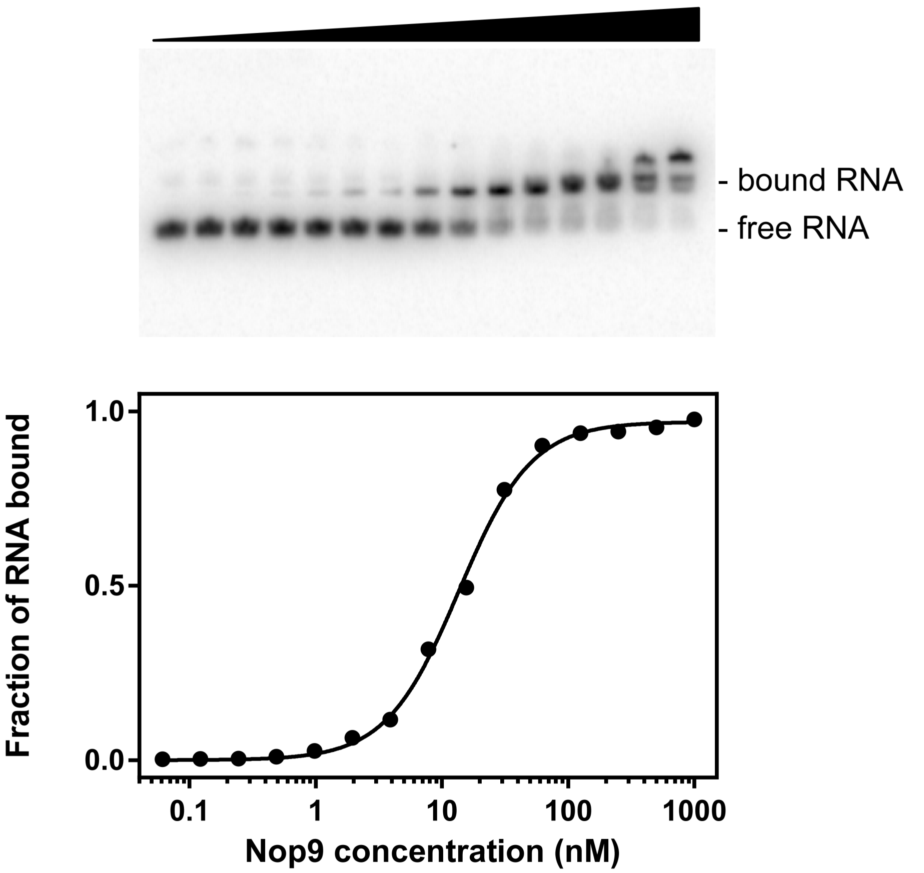


**Supplementary Figure 3.** Representative EMSA binding data for Nop9 binding to ITS1 Subdomain A $\Delta 5'_{1\text{-}6}$, $\Delta 3'$. A representative gel (top) with corresponding binding curve (bottom) is shown. Protein concentrations are serially diluted 2 fold from 1000 nM.

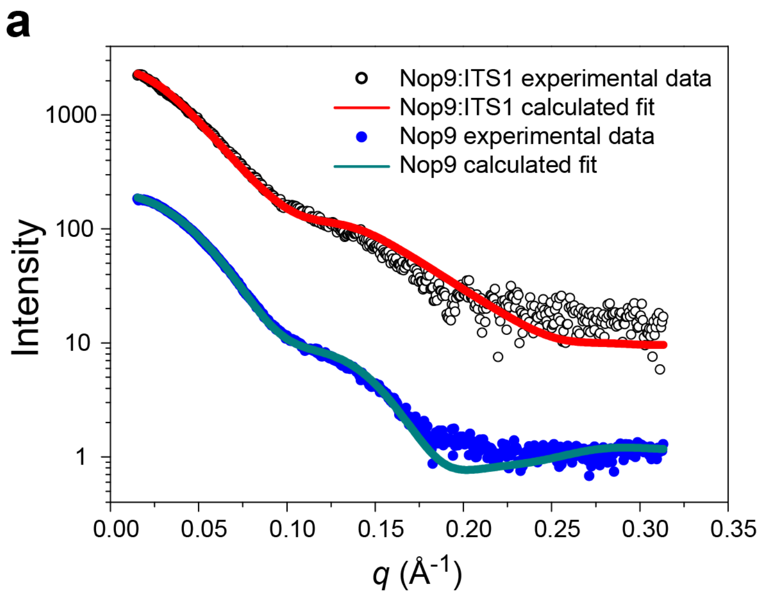


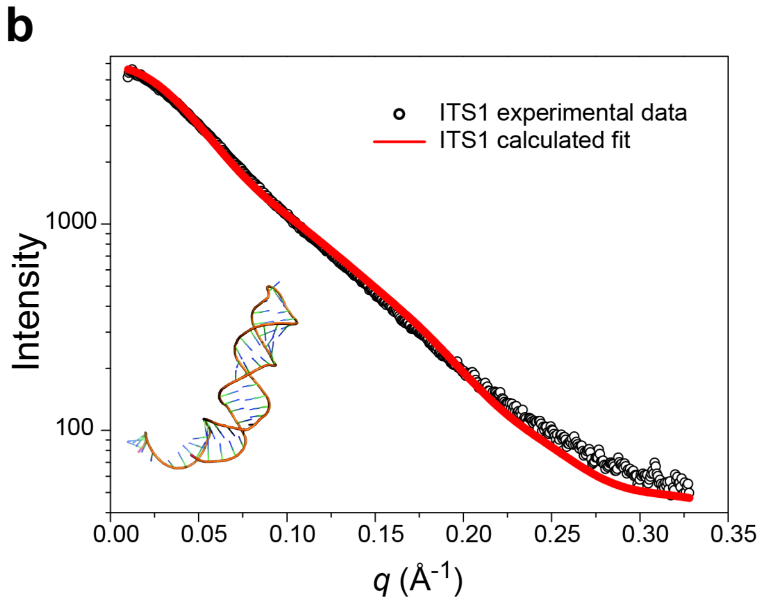


**Supplementary Figure 4.** SAXS experimental and calculated scattering curves. (a) Superimposed experimental and calculated scattering curves for Nop9 alone and a Nop9:ITS1 RNA complex calculated by rigid-body docking. The $\chi^2$ for the calculated fit of Nop9 alone and its corresponding data is 2.9, and the $\chi^2$ for the calculated fit of Nop9:ITS1 and its corresponding data is 2.0. (b) Superimposed experimental and calculated scattering curves for ITS1 rRNA (7-38_184-206). The $\chi^2$ for the calculated fit of ITS1 rRNA alone and its corresponding data is 3.8. The predicted RNA model is shown in the inset. This figure was generated using the FoXS web server[5,6].

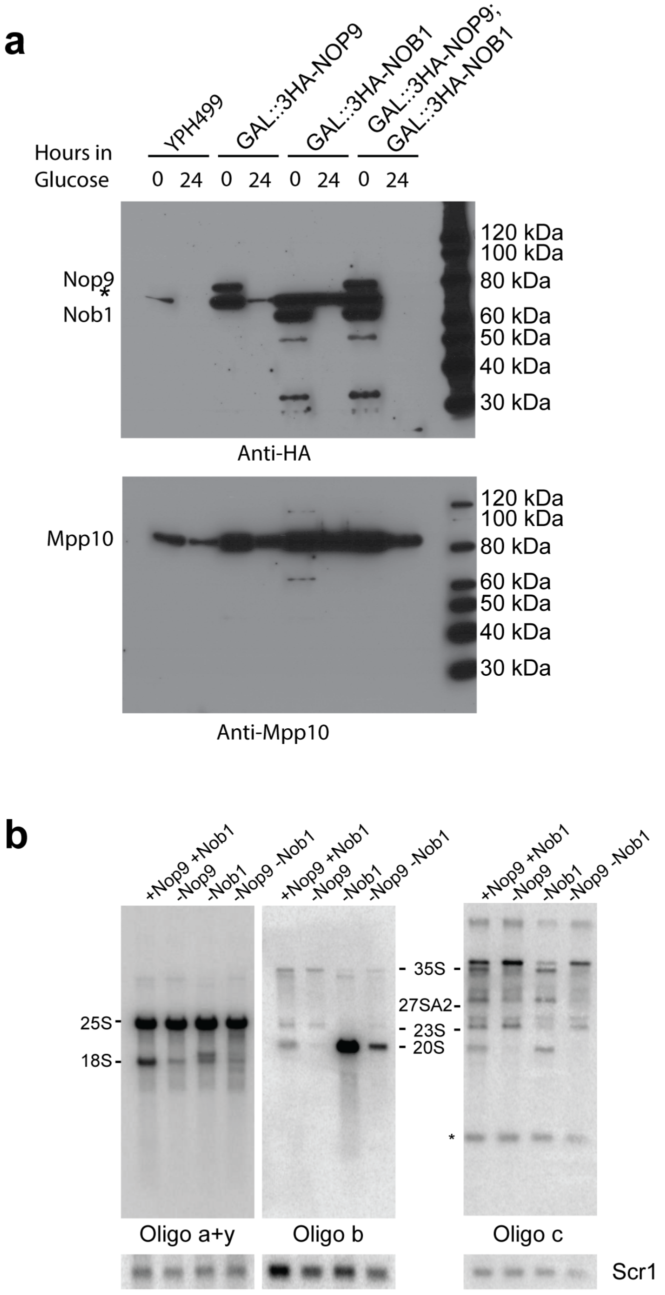


**Supplementary Figure 5.** *In vivo* effects of depletion of Nop9 and/or Nob1. (a) Western blot confirming depletion of HA-tagged Nop9 and/or Nob1 protein expression. The positions of HA-tagged Nop9 and Nob1 proteins are indicated in the top blot. * denotes a 50 kDa anti-HA tag cross-reactive band observed in all strains. The blot was stripped and probed with an anti-Mpp10 antibody as a loading control. Molecular weight markers are shown at the right of each blot. (b) Northern blot analysis of Nop9/Nob1 double depletion. Full representative northern blots detecting precursor and mature rRNA in total RNA as presented in Fig. 5c. An oligo complementary to Scr1 was used as a loading control (indicated with * in the full northern blot probed with oligo c).

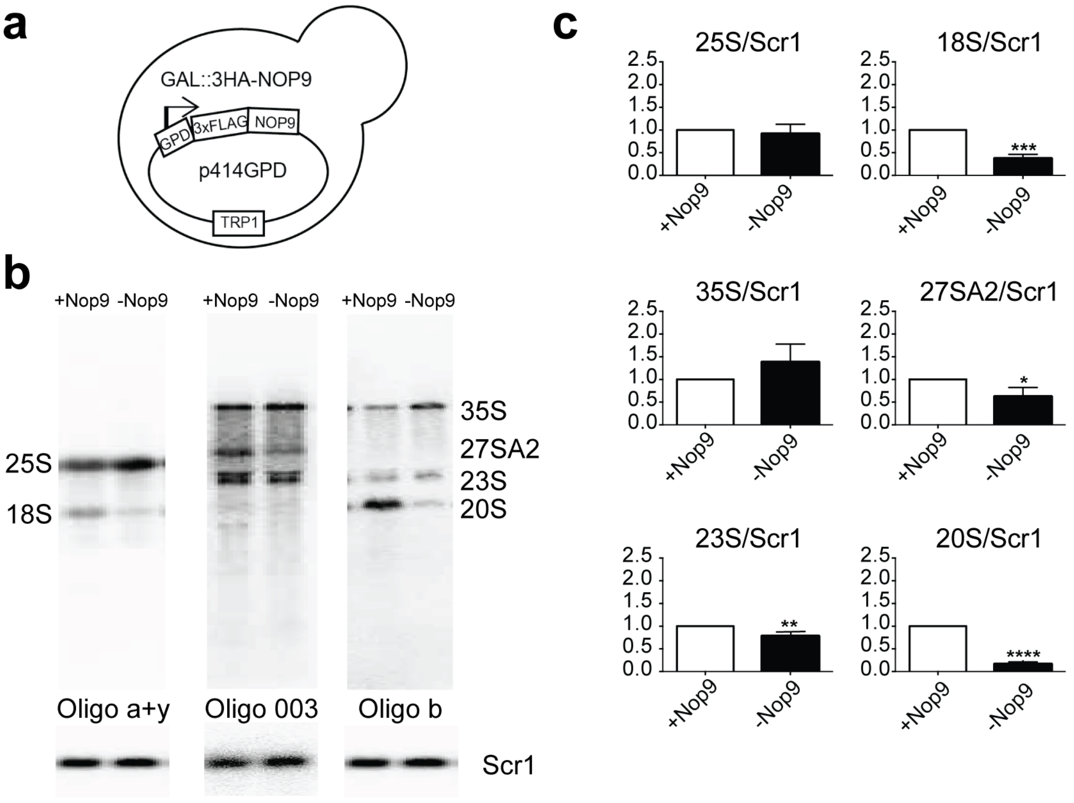


**Supplementary Figure 6.** Depletion of Nop9 reduces 20S pre-rRNA levels. (a) Schematic drawing of the yeast strain used for testing the effects of Nop9 depletion. Chromosomal Nop9 expression was placed under the control of the inducible *GAL* promoter and sequences encoding triple-HA tags were inserted. The *GAL::3HA-NOP9* yeast strain was transformed with the empty vector (-Nop9) or Nop9 (+Nop9) in p414GPD-3xFLAG plasmid. (b) Representative northern blots detecting precursor and mature rRNA in yeast depleted of Nop9 or expressing wild type Nop9. Total RNA was extracted from yeast bearing a plasmid expressing wild type Nop9 (+Nop9) or empty vector (-Nop9) after depletion of endogenous Nop9 in glucose for 72 hours at 17 °C. The pre-rRNA intermediates and mature rRNAs were detected using a series of oligonucleotide probes, 003, a, b and y, that are indicated in Fig. 1a. Oligo 003 was used to detect 35S, 27SA2, and 23S pre-rRNAs, oligo b was used to detect 20S pre-rRNA, and oligos a + y were used to detect 25S and 18S rRNAs. (c) Plot of the intensities of the mature rRNAs and pre-rRNA intermediates detected in panel b relative to Scr1, the loading control. Bar graphs created in GraphPad PRISM plot the means calculated from three biological replicate experiments with error bars representing the standard error of the mean. The significance of the levels of mature and pre-rRNAs in Nop9-depleted yeast (-Nop9) compared with +Nop9 was assessed by an unpaired t-test, and p-values are indicated (* indicates $p \leq 0.05$, ** indicates $p \leq 0.01$, **** indicates $p \leq 0.0001$, non-significant differences have p-values > 0.05). The analysis of 35S rRNA and 23S pre-rRNA using oligo 003 is shown, but similar results are obtained when analyzing the blot probed with oligo b.

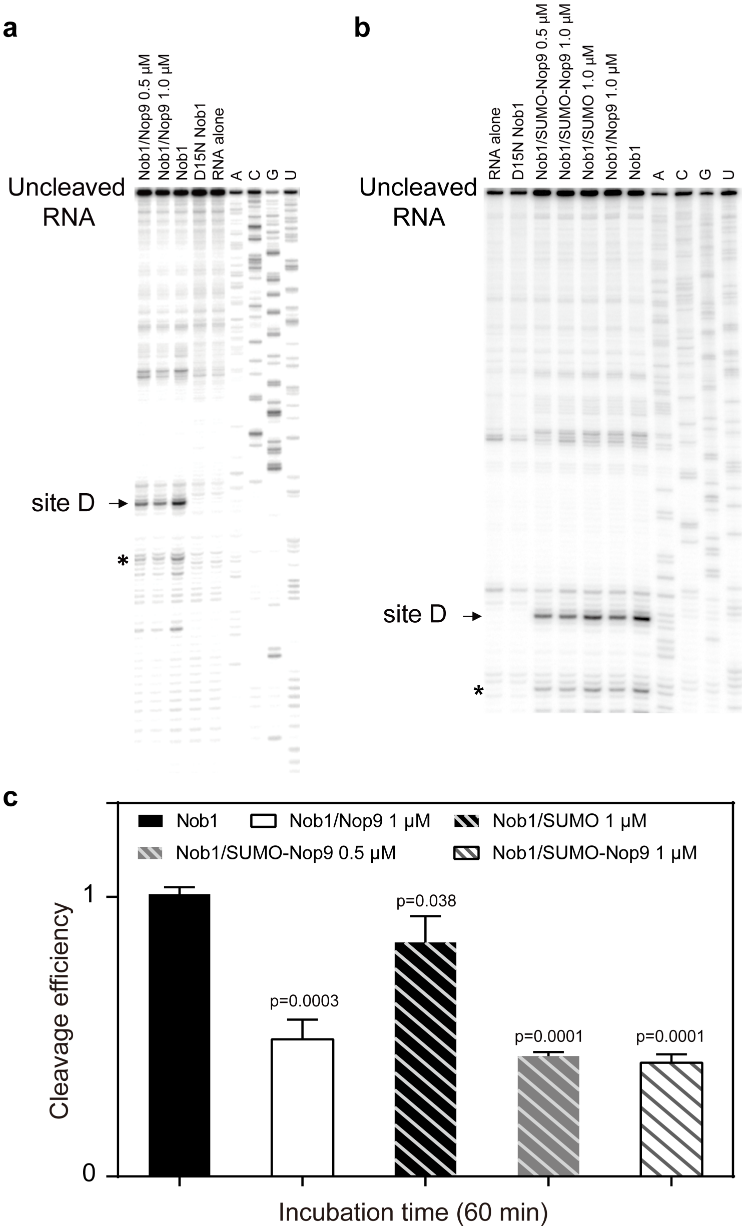


**Supplementary Figure 7.** Primer extension analysis demonstrating Nob1 cleavage at site D and inhibition by Nop9. (a) Representative full-length gel of primer extension analysis of Nob1 cleavage products. (b) Inhibition of Nob1 cleavage by SUMO-Nop9 fusion protein or Nop9 protein. For both panels, an arrow indicates a major product with cleavage at site D. An asterisk indicates a minor product at an alternative cleavage site. Sequencing reactions are shown in the right lanes. Three technical replicates were conducted. (c) Nob1 cleavage efficiencies in the presence of Nop9 or SUMO-Nop9. Cleavage efficiencies were calculated as the ratio of site D cleavage product to total RNA. The efficiencies of cleavage by Nob1 or Nob1/SUMO were set to 1. Mean cleavage efficiencies ± S.E.M. were 24.4 ± 0.36% for Nob1 and 20.2 ± 1.3% for Nob1/SUMO. Bar graphs, created in GraphPad PRISM, plot the means calculated from three technical replicate experiments with error bars representing the S.E.M. The significance of the Nob1 cleavage efficiencies in the presence of Nop9 relative to Nob1 cleavage alone or SUMO-Nop9 relative to SUMO alone was assessed by an unpaired, two-sided t-test, and p-values are indicated.

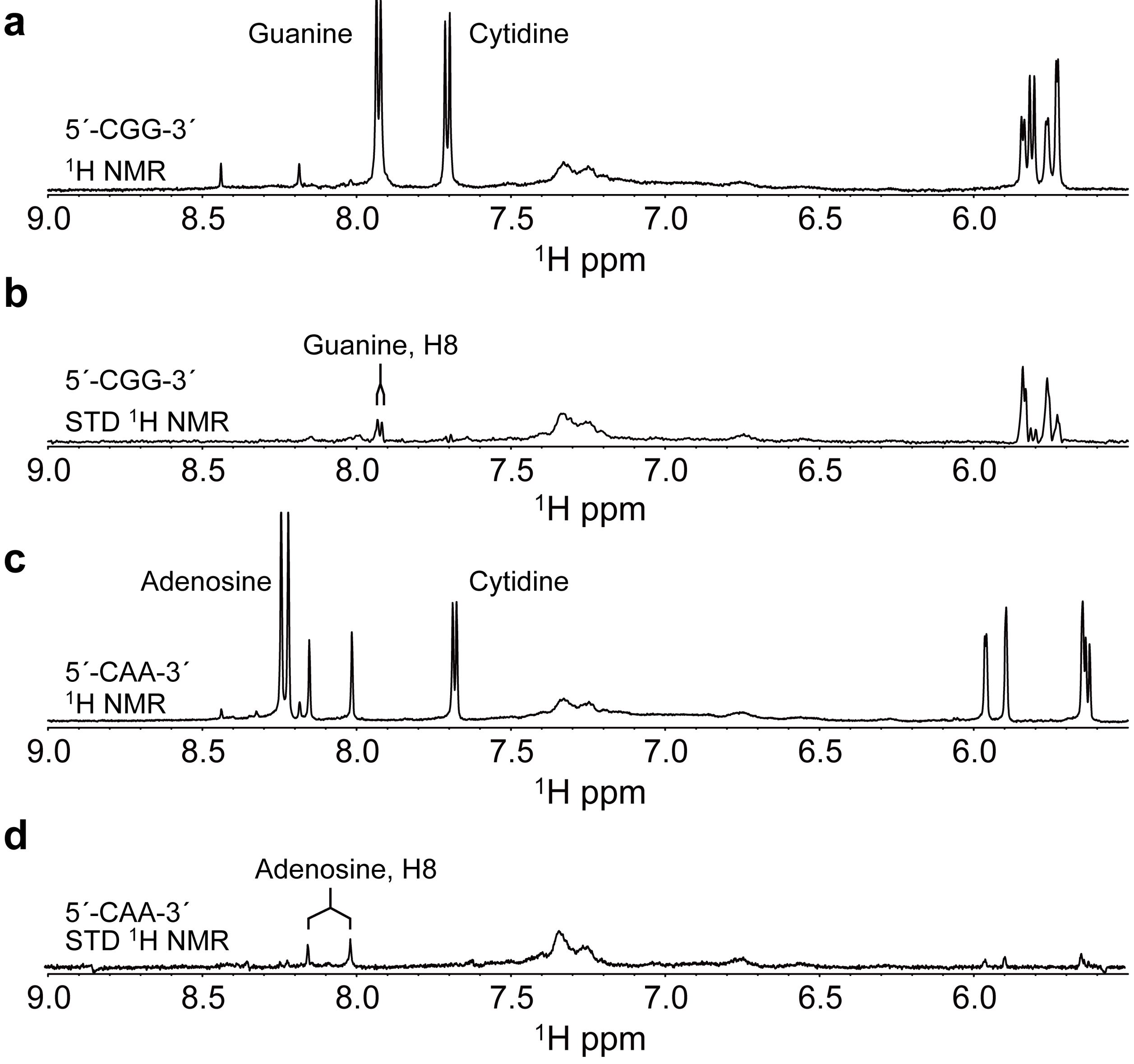


**Supplementary Figure 8.** Saturation-Transfer Difference (STD) NMR of short RNA fragments with Nop9. (a) Reference $^{1}$H spectrum of 5´-CGG-3´ with Nop9 and (c) STD spectrum. (c) Reference $^{1}$H spectrum of 5´-CAA-3´ with Nop9 and (d) STD spectrum.

**Supplementary Table 1. Nop9 binds to the ITS1 RNA stem loop**

| RNA | $K_d$ (nM)[a] | $K_{rel}$[b] | *p*-value[c] |
|---|---|---|---|
| ITS1 Subdomain A | 2.4 ± 0.3 | 1 | N/A |
| ITS1 Subdomain B | 105.0 ± 2.9 | 44 | 0.0001 |
| ITS1 Subdomain C | 74.4 ± 4.1 | 31 | 0.0001 |
| Subdomain A $\Delta 5'_{1\text{-}16}$ | >1000 | | N/A |
| Subdomain A 1-16 | >1000 | | N/A |
| Subdomain A $\Delta 5'_{1\text{-}6}$ | 1.7 ± 0.4 | 0.7 | 0.23 |
| Subdomain A $\Delta 3'$ | 15.7 ± 2.6 | 6.5 | 0.0071 |
| Subdomain A $\Delta 5'_{1\text{-}6}$, $\Delta 3'$ | 11.8 ± 0.4 | 4.9 | 0.0001 |

[a]$K_d$'s are mean ± SEM for three technical replicates with the exception of binding to Subdomain A $\Delta 5'_{1\text{-}6}$, $\Delta 3'$, which was for four technical replicates. [b]$K_{rel}$ was calculated relative to the $K_d$ for Nop9 binding to ITS1 Subdomain A. [c]The significance of Nop9 binding affinity to the indicated RNAs relative to Nop9 binding to ITS1 Subdomain A was assessed by an unpaired, two-sided t-test, and *p*-values are indicated.

**Supplementary Table 2. Nop9 binding to ITS1 RNA is modestly sensitive to salt concentration**

| RNA | $K_d$ (nM)[a] | $K_{rel}$[b] | *p*-value[c] |
|---|---|---|---|
| Subdomain A $\Delta 5'_{1\text{-}6}$, 150 mM NaCl | 1.7 ± 0.4 | 1 | N/A |
| Subdomain A $\Delta 5'_{1\text{-}6}$, 250 mM NaCl | 4.8 ± 1.0 | 2.8 | 0.083 |
| Subdomain A $\Delta 5'_{1\text{-}6}$, 500 mM NaCl | 15.6 ± 2.4 | 9.2 | 0.0055 |

[a]$K_d$'s are mean ± SEM for three technical replicates. [b]$K_{rel}$ was calculated relative to the $K_d$ for Nop9 binding to ITS1 Subdomain A $\Delta 5'_{1\text{-}6}$ in buffer containing 150 mM NaCl. [c]The significance of Nop9 binding affinity at the indicated NaCl concentration relative to Nop9 binding to ITS1 Subdomain A $\Delta 5'_{1\text{-}6}$ in buffer containing 150 mM NaCl was assessed by an unpaired, two-sided t-test, and *p*-values are indicated.

**Supplementary Table 3. Nop9 binds to the base of the ITS1 RNA stem loop**

| RNA | $K_d$ (nM)[a] | $K_{rel}$[b] | *p*-value[c] |
|---|---|---|---|
| Subdomain A Δ5′$_{1-6}$, Δ3′ | 11.8 ± 0.4 | 1 | N/A |
| Subdomain A Δ5′$_{1-12}$, Δ3′ | 18.2 ± 1.9 | 1.5 | 0.0119 |
| Subdomain A Δ5′$_{1-15}$, Δ3′ | 346 ± 40 | 29.3 | 0.0002 |
| UUU(7-9)GGG Subdomain A Δ5′$_{1-6}$, Δ3′ | 6.6 ± 0.3 | 0.6 | 0.0002 |
| AAU(10-12)CCC Subdomain A Δ5′$_{1-6}$, Δ3′ | 8.5 ± 0.6 | 0.7 | 0.0050 |
| AAU(13-15)CCC Subdomain A Δ5′$_{1-6}$, Δ3′ | 27.6 ± 3.7 | 2.3 | 0.0039 |
| UUU(16-18)GGC, AA(205-206)GC Subdomain A Δ5′$_{1-6}$, Δ3′ | 103 ± 12 | 8.7 | 0.0003 |
| U16G Subdomain A Δ5′$_{1-6}$, Δ3′ | 47.7 ± 1.8 | 4.0 | 0.0001 |
| U16A Subdomain A Δ5′$_{1-6}$, Δ3′ | 62.3 ± 4.9 | 5.3 | 0.0001 |
| U16C Subdomain A Δ5′$_{1-6}$, Δ3′ | 200.9 ± 27.3 | 17.0 | 0.0001 |
| UU(17-18)GC, AA(205-206)GC Subdomain A Δ5′$_{1-6}$, Δ3′ | 45.8 ± 5.9 | 3.9 | 0.0010 |
| AA(205-206)GG Subdomain A Δ5′$_{1-6}$, Δ3′ | 33.2 ± 1.5 | 2.8 | 0.0001 |
| UUG(17-19)AAC, CAA(204-206)GUU Subdomain A Δ5′$_{1-6}$, Δ3′ | 14.3 ± 1.4 | 1.2 | 0.1047 |
| AAA(20-22)CCC, UUU(201-203)GGG Subdomain A Δ5′$_{1-6}$, Δ3′ | 7.9 ± 0.4 | 0.7 | 0.0011 |
| AUUU(27-30)GCGC, AAAA(194-197)GCGC Subdomain A Δ5′$_{1-6}$, Δ3′ | 20.0 ± 1.5 | 1.7 | 0.0017 |

[a]$K_d$'s are mean ± SEM for three technical replicates with the exception of binding to Subdomain A Δ5′$_{1-6}$, Δ3′, which was for four technical replicates.. [b]$K_{rel}$ is calculated relative to the $K_d$ for Nop9 binding to ITS1 Subdomain A Δ5′$_{1-6}$, Δ3′. Shading corresponds to Figure 3: $K_{rel} > 3$, dark orange; $2 < K_{rel} < 3$, light orange; $1 < K_{rel} < 2$, dark gray; $K_{rel} \leq 1$, light gray. [c]The significance of Nop9 binding affinity to the indicated RNAs relative to Nop9 binding to Subdomain A Δ5′$_{1-6}$, Δ3′ was assessed by an unpaired, two-sided t-test, and *p*-values are indicated.

**Supplementary Table 4. Radius of gyration ($R_g$) values at different sample concentrations**

| Nop9 | | ITS1 RNA | | Nop9:ITS1 RNA | |
|---|---|---|---|---|---|
| Concentration (mg/ml) | $R_g$ (Å)[a] | Concentration (mg/ml) | $R_g$ (Å) | Concentration (mg/ml) | $R_g$ (Å) |
| 1.27 | 32.67 ± 0.01 | 0.67 | 28.64 ± 0.19 | 0.88 | 39.53 ± 0.64 |
| 2.88 | 32.66 ± 0.02 | 1.33 | 26.73 ± 0.01 | 1.43 | 40.51 ± 0.55 |
| 6.13 | 34.45 ± 0.03 | 2.67 | 26.33 ± 0.33 | 2.37 | 40.73 ± 0.20 |

[a]$R_g$'s are mean ± SD for three technical replicates.

**Supplementary Table 5. Primers used in this study**

| Sequence description | Sequence (5′ to 3′)[a] |
|---|---|
| 5′ ITS1 1-212 | GAAATTAATACGACTCACTATAGG<br>AAGAAATTTAATAATTTTGAAAATGGATTTTTTTG |
| 3′ ITS1 1-212 | GTTGTATTGAAACGGTTTTAATTGTCCTATAACAAAAGCACAG |
| ITS1 77-140<br>Subdomain C<br>sense strand | GAAATTAATACGACTCACTATAGG<br>AGAGATGGAGAGTCCAGCCGGGCCTGCGCTTAAGTGCGCGGTCT<br>TGCTAGGCTTGTAAGTTTCT |
| ITS1 39-77_140-183<br>Subdomain B<br>sense strand | GAAATTAATACGACTCACTATAGG<br>TGGCAAGAGCATGAGAGCTTTTACTGGGCAAGAAGACAA_<br>TTTCTTGCTATTCCAAACGGTGAGAGATTTCTGTGCTTTTGTTA |
| ITS1 1-38_184-212<br>Subdomain A<br>sense strand | GAAATTAATACGACTCACTATAGG<br>AAGAAATTTAATAATTTTGAAAATGGATTTTTTTGTTT_*TA*_<br>TAGGACAATTAAAACCGTTTCAATACAAC |
| ITS1 17-38_184-206<br>sense strand | GAAATTAATACGACTCACTATAGG<br>TTGAAAATGGATTTTTTTGTTT_*TA*_<br>TAGGACAATTAAAACCGTTTCAA |
| ITS1 7-38_184-206<br>sense strand | GAAATTAATACGACTCACTATAGG<br>TTTAATAATTTTGAAAATGGATTTTTTTGTTT_*TA*_<br>TAGGACAATTAAAACCGTTTCAA |
| ITS1 10-38_184-206<br>sense strand | GAAATTAATACGACTCACTATAGG<br>AATAATTTTGAAAATGGATTTTTTTGTTT_*TA*_<br>TAGGACAATTAAAACCGTTTCAA |
| ITS1 13-38_184-206<br>sense strand | GAAATTAATACGACTCACTATAGG<br>AATTTTGAAAATGGATTTTTTTGTTT_*TA*_<br>TAGGACAATTAAAACCGTTTCAA |
| ITS1 16-38_184-206<br>sense strand | GAAATTAATACGACTCACTATAGG<br>TTTGAAAATGGATTTTTTTGTTT_*TA*_<br>TAGGACAATTAAAACCGTTTCAA |
| ITS1 7-38_184-206<br>UUU(7-9)GGG<br>sense strand | GAAATTAATACGACTCACTATAGG<br>*GGG*AATAATTTTGAAAATGGATTTTTTTGTTT_*TA*_<br>TAGGACAATTAAAACCGTTTCAA |
| ITS1 7-38_184-206<br>AAU(10-12)CCC<br>sense strand | GAAATTAATACGACTCACTATAGG<br>TTT*CCC*AATTTTGAAAATGGATTTTTTTGTTT_*TA*_<br>TAGGACAATTAAAACCGTTTCAA |
| ITS1 7-38_184-206<br>AAU(13-15)CCC<br>sense strand | GAAATTAATACGACTCACTATAGG<br>TTTAAT*CCC*TTTGAAAATGGATTTTTTTGTTT_*TA*_<br>TAGGACAATTAAAACCGTTTCAA |
| ITS1 7-38_184-206<br>UUU(16-18)GGC<br>sense strand | GAAATTAATACGACTCACTATAGG<br>TTTAATAAT*GGC*GAAAATGGATTTTTTTGTTT_*TA*_<br>TAGGACAATTAAAACCGTTTC*GC* |
| ITS1 7-38_184-206 U16G<br>sense strand | GAAATTAATACGACTCACTATAGG<br>TTTAATAAT*G*TTGAAAATGGATTTTTTTGTTT_*TA*_<br>TAGGACAATTAAAACCGTTTCAA |
| ITS1 7-38_184-206<br>UU(17-18)GC<br>sense strand | GAAATTAATACGACTCACTATAGG<br>TTTAATAATT_*GC*_TGAAAATGGATTTTTTTGTTT_*TA*_<br>TAGGACAATTAAAACCGTTTC*GC* |

| | |
|---|---|
| ITS1 7-38_184-206<br>UUG(17-19)AAC<br>sense strand | GAAATTAATACGACTCACTATAGG<br>TTTAATAATT*AAC*AAAATGGATTTTTTTGTTT_*TA*_<br>TAGGACAATTAAAACCGTTT*GTT* |
| ITS1 7-38_184-206<br>AAA(20-22)CCC<br>sense strand | GAAATTAATACGACTCACTATAGG<br>TTTAATAATTTTG*CCC*ATGGATTTTTTTGTTT_*TA*_<br>TAGGACAATTAAAACCG*GGG*CAA |
| ITS1 7-38_184-206<br>AUUU(27-30)GCGC<br>sense strand | GAAATTAATACGACTCACTATAGG<br>TTTAATAATTTTGAAAATGG*GCGC*TTTTGTTT_*TA*_<br>TAGGACAATT*GCGC*CCGTTTCAA |
| 5′ ITS1 -164<br>sense strand | GAAATTAATACGACTCACTATAGG<br>CCGCCCGTCGCTAGTACCGATTG |
| ITS1_62-84_R | CCATCTCTTGTCTTCTTGCCCAG |
| Oligo 003 | TGCTTACCTCTGGGCC |
| Oligo c | ATGAAAACTCCACAGTG |
| Oligo a | CATGGCTTAATCTTTGAGAC |
| Oligo y | GCCCGTTCCCTTGGCTGTG |
| Oligo b | GCTCTTTGCTCTTGCC |
| Oligo Scr1 | CGTGTCTAGCCGCGAGGAAGGATTTGTTCC |
| Nop9 depletion strain<br>Forward primer | GTTACAACTAGTATACTGTGAACGACGCTGAAACCTTCACGGAA<br>AAACCACATTATTGTTATTGAATTCGAGCTCGTTTAAAC |
| Nop9 depletion strain<br>Reverse primer | GGGTTCAAATTCGTCCTTTCTCTGTTTGTCTTGGTGTCTTCTGCCT<br>CTTGTTTTAGTCTTTCCCATGCACTGAGCAGCGTAATCTG |
| Nob1 depletion strain<br>Forward primer | CGGCGATCATAGTTCAGTATTTTTCTAAAGTTTCTTTAAAGGAGC<br>ATAATGAATTCGAGCTCGTTTAAAC |
| Nob1 depletion strain<br>Reverse primer | GGGGTGGCATCCAATATCAACGCCCTTACATGTGCGGTTTGGTTT<br>TCGGTCATGCACTGAGCAGCGTAATCTG |

[a] For sense strands, the first line indicates the T7 promoter sequence. _*TA*_ denotes the truncated ITS1 loop. Mutated bases are italicized and underlined.

**Supplementary References**


1 Thompson, J. D., Higgins, D. G. & Gibson, T. J. CLUSTAL W: improving the sensitivity of progressive multiple sequence alignment through sequence weighting, position-specific gap penalties and weight matrix choice. *Nucleic Acids Res* **22**, 4673-4680 (1994).

2 Abbasi, N. *et al.* APUM23, a nucleolar Puf domain protein, is involved in pre-ribosomal RNA processing and normal growth patterning in Arabidopsis. *Plant J* **64**, 960-976, doi:10.1111/j.1365-313X.2010.04393.x (2010).

3 Huang, T., Kerstetter, R. A. & Irish, V. F. APUM23, a PUF family protein, functions in leaf development and organ polarity in Arabidopsis. *J Exp Bot* **65**, 1181-1191, doi:10.1093/jxb/ert478 (2014).

4 Zhang, C. & Muench, D. G. A Nucleolar PUF RNA-binding Protein with Specificity for a Unique RNA Sequence. *J Biol Chem*, doi:10.1074/jbc.M115.691675 (2015).

5 Schneidman-Duhovny, D., Hammel, M., Tainer, J. A. & Sali, A. Accurate SAXS profile computation and its assessment by contrast variation experiments. *Biophys J* **105**, 962-974, doi:10.1016/j.bpj.2013.07.020 (2013).

6 Schneidman-Duhovny, D., Hammel, M. & Sali, A. FoXS: a web server for rapid computation and fitting of SAXS profiles. *Nucleic Acids Res* **38**, W540-544, doi:10.1093/nar/gkq461 (2010).
